# Understanding Women’s Knowledge, Awareness, and Perceptions of STIs/STDs in Asia: A Scoping Review

**DOI:** 10.3390/healthcare11192643

**Published:** 2023-09-28

**Authors:** Wandeep Kaur, Vimala Balakrishnan, Ian Ng Zhi Wei, Annabel Yeo Yung Chen, Zhao Ni

**Affiliations:** 1 Faculty of Information Science & Technology, Universiti Kebangsaan Malaysia, Bangi 43600, Malaysia; wandeep@ukm.edu.my; 2 Faculty of Computer Science and Information Technology, Universiti Malaya, Kuala Lumpur 50603, Malaysia; vimala.balakrishnan@um.edu.my; 3 Faculty of Medicine, Universiti Malaya, Kuala Lumpur 50603, Malaysia; u2104667@siswa.um.edu.my (I.N.Z.W.); s2101794@siswa.um.edu.my (A.Y.Y.C.); 4 School of Nursing, Yale University, New Haven, CT 06477, USA

**Keywords:** HIV, sexually transmitted infection, sexually transmitted disease, women, Asia, knowledge, awareness, perception

## Abstract

Objective: This study aimed to conduct a scoping review to collect current literature on the knowledge, awareness, and perception (KAP) of sexually transmitted infections/diseases (STIs/STDs) among women in Asia. Methodology: The PRISMA-Scoping methodology was used in this study to extract papers from four databases published between 2018 and 2022. Sixty-eight articles were included after screening and elimination. Results: The studies on KAP of STIs/STDs among women were largely undertaken in Southeast Asia (Indonesia, Malaysia, and Vietnam) and South Asia (India, Pakistan, and Bangladesh). Regardless of the specific cohort of women studied, research indicates consistently low levels of knowledge and awareness across Asia. This trend seems to be more prevalent among female commercial sex workers, women with lower educational levels, and those in poorer socioeconomic positions. In South Asia, cultural, sociological, economic, and gender inequalities, particularly male domination, all have a significant impact on KAP levels. Conclusion: As education is a major factor that influences health behaviour, this review underscores the need to allocate more resources to educational initiatives, particularly targeting vulnerable groups such as sex workers, transgender women, pregnant women, and rural housewives. This strategic focus may contribute significantly to preventing STIs/STDs, particularly in less developed regions/countries.

## 1. Introduction

The prevalence of human immunodeficiency virus (HIV) among women in Asia is significantly elevated, which poses substantial risks to their health and well-being [1]. Women living with HIV in Asia may be subject to social stigma and discrimination, both of which can have a detrimental effect not only on their mental health but also on their overall quality of life [2,3]. HIV damages the immune system, making patients prone to infections and other disorders. In 2021, the Joint United Nations Programme on HIV/AIDS reported 2.2 million HIV-positive Asians, with women accounting for over half of all new infections. Sexually transmitted infections/diseases (STIs/STDs), such as hepatitis B, chlamydia, and gonorrhoea, are also major health risks for Asian women and can lead to infertility and increase the risk of HIV infection [4]. HIV and various STIs/STDs are mostly transferred through sexual activity, including vaginal, anal, and oral intercourse [5]. The predominant mode of transmission described above makes individuals who engage in unprotected sexual activity or have several sexual partners particularly vulnerable to both HIV and STIs/STDs. Consequently, it is suggested that a lack of sufficient knowledge about STIs/STDs may potentially lead to insufficient awareness and understanding of HIV. Thus, this research postulates a correlation between inadequate understanding and awareness of HIV and a corresponding lack of knowledge and awareness of STIs/STDs.

There is a correlation between socioeconomic disparity and STI/STD/HIV awareness. Especially in low- and middle-income countries (LMICs), a lack of information and health education is one of the key causes of increasing HIV infection rates in women of reproductive age (15–49 years) [6]. The third Sustainable Development Goal (SDG 3) aims to achieve all three of the following by the year 2030: zero new HIV infections, zero discrimination, and zero deaths caused by AIDS. To achieve this objective, it is essential for researchers to understand the degree of knowledge, awareness, and perceptions (KAP) held by Asian women in order to identify the most vulnerable groups in need of immediate action.

In this paper, we adhered to the following definitions of KAP. Knowledge refers to a person’s knowledge of fundamental facts, such as the modes of transmission and symptoms, which are normally learned through formal schooling. Awareness refers to a person’s comprehension of the cultural context, including the prevention, testing, treatment, and stigma associated with STI/STD [7]. Perception is defined as an individual’s attitude or viewpoint, which can be positive or negative [8]. 

Existing review articles have focused on a single cohort of women, i.e., women living with HIV [9], female sex workers [10,11], and transgender women [12]. Two review studies on knowledge of HIV were conducted by analysing publications from India [13,14], while [15] conducted a review of studies published on cervical cancer screening among women in Africa. Another review [9] looked into the psychological and social impact of women living with HIV in LMIC countries. To the extent that we are aware, no review of the literature on the KAP of women from all cohorts in Asia has been carried out to date, nor has any other kind of literature synthesis been attempted. This review looks to bridge this gap by understanding women’s KAP of STIs/STDs in Asian countries. 

This review differs from existing reviews in several ways: (i) by taking a wider pool of studies into consideration (2018–2022), thus covering the level of KAP among women throughout Asia during the last 5 years; (ii) by targeting Asian countries, many of which are developing nations and plagued with stigma and cultural differences with regard to HIV; and (iii) by focusing on women of reproductive ages (15–49 years) from all cohorts, including transgender women and female sex workers. This review constitutes an integral component of a comprehensive study centred around the assessment of knowledge, awareness, attitudes, perceptions, and risky sexual conduct across diverse cohorts. Specifically, the current review is concentrated on investigating the KAP pertaining to STIs/STDs, encompassing HIV, among women residing in Asian regions. In contrast, the research conducted by [16] encompassed a broader analysis of knowledge, attitudes, and perceptions concerning STIs in a general context, but limited to the geographical scope of Southeast Asian nations.

## 2. Materials and Methods 

This review is a component of a comprehensive study involving countries in Asia. The five-stage approach for scoping reviews was adopted in accordance with the PRISMA Extension for Scoping Reviews [17,18]. The stages are elaborated in the subsequent sections:

### 2.1. Research Questions 

The following four research questions (RQs) were formulated to support a synthesis of knowledge from the extant literature on the barriers to women’s access to HIV- and STI/STD-related information and health services.

RQ1—What are the studies targeting KAP among women in Asia?

RQ2—What are the levels of KAP among women in Asia?

RQ3—What are the susceptible groups among women requiring immediate action?

RQ4—What are the risky behaviours women engage in due to lack of KAP?

### 2.2. Search Strategy

The main strategy used in the larger review study conducted will be discussed in this sub-section. Articles published between 2018 to 2022 were downloaded from four academic databases (PubMed, Web of Science (WoS), Scopus, and the Cumulated Index to Nursing and Allied Health Literature (CINALH)). The search was limited to studies conducted in Asia. The search strings used were as follows:

– (sex* transm* OR STI OR STD) AND (know* OR beh* OR aware* OR attitude* OR perce* OR stigma* OR risk*)
– (HIV OR HPV) AND (know* OR beh* OR aware* OR attitude* OR perce* OR stigma* OR risk*)

### 2.3. Study Selection

Using the above search terms, 31,021 articles were downloaded, which were then sorted through based on the inclusion and exclusion criteria stated below. As a result, 29,206 articles were taken off the list during Phase 1 of the screening stage.

Inclusion criteria:

- Published articles, including those in press.
- Focus on knowledge, awareness, perception, and risky behaviour.
- HIV and STI/STD (any).
- Valid research methodology (i.e., empirical, experiments, content analysis, etc.).
- Articles in English.
- Location of study = Asia.
- Cohort = women (including girls, transgender women, and female sex workers).

Exclusion criteria:

- Pre-prints, along with book chapters, conference proceedings, letters to editors, viewpoints, etc.
- Focus on stigmatization, prevalence, coping strategies, and effects of intervention strategies on these aspects.
- Clinical studies.
- HPV, HPV vaccination studies.
- Studies not related to HIV or STI/STD.

**Figure 1:**
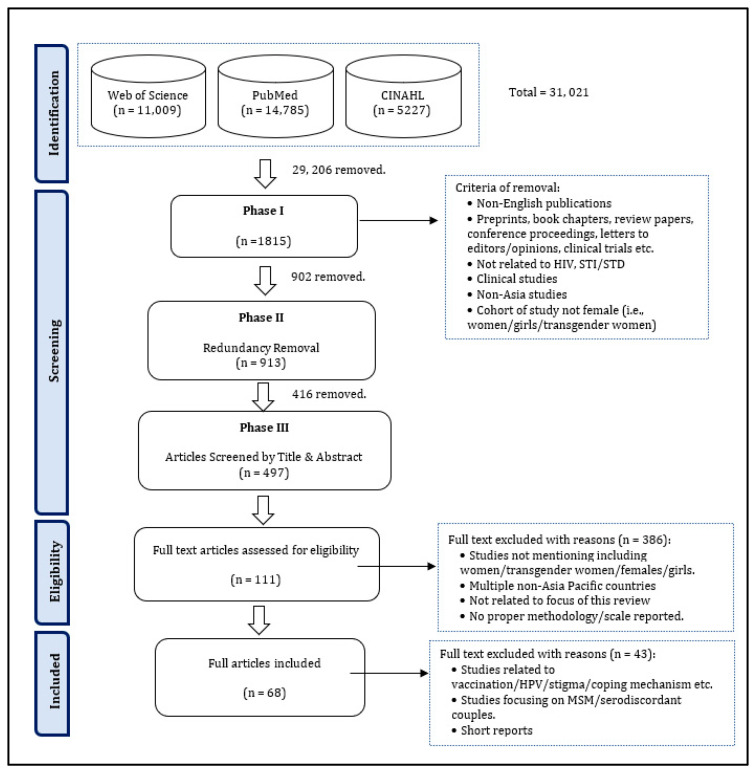
PRISMA-SCR flowchart.

After removing duplicates, 913 items remained. Three reviewers assessed the titles and abstracts for suitability. A fourth reviewer was appointed to resolve disputes. EndNote was used to manage the review process as well as to eliminate any possible duplication. This review selected 68 papers. Figure 1 shows the PRISMA-Scoping diagram for these steps.

### 2.4. Charting the Data 

This stage examined all 68 articles and extracted data for the four RQs. Authors, year of publication, country of study, aim, cohort, focus (KAP), instruments, and important results were retrieved. This was performed by three reviewers, and the results were double-checked by a fourth reviewer to reduce the likelihood of errors. It needs to be noted that as long as one of the elements of KAP was being investigated, the published study was included in the pool of the review. The comprehensive analysis is available in Supplementary Materials Table S1. Tables and charts describing this review’s findings are presented in the next section. 

## 3. Results and Discussion 

### 3.1. Study Characteristics 

This section addresses RQ1, which focused on the current state of academic research focusing on KAP and STD/STI in Asia. Table 1 shows the descriptive data for the studied papers, ranging from 4 published in 2022 to 17 in 2020 and 2021. Based on the data, Southeast Asia recorded the highest number of studies conducted, with Indonesia (*n* = 8), Malaysia (*n* = 4), Vietnam (*n* = 3), and Thailand (*n* = 3) emerging as the top four countries, followed by Cambodia (*n* = 2) and one study in Myanmar [19]. China (*n* = 11) produced the highest number in East Asia, which included one multi-country study [20]. Iran (*n* = 11) published the most papers in the western Asian region, with one paper published from Lebanon [7]. 

Female sex workers (*n* = 19) and transgender women (*n* = 15) dominated the articles. Most female sex worker studies (*n* = 6) and transgender women (*n* = 4) studies were conducted in China. Lastly, sociodemographic profiles focused more on education (*n* = 22), socio-economic/income (*n* = 15), age (*n* = 14), marital status (*n* = 11), and occupation (*n* = 7). 

### 3.2. Levels of Knowledge, Awareness, and Perception (KAP) about HIV, STI/STD among Women in Asia 

Papers that specifically provided details to answer RQ2 are discussed in this section. Table 2 summarises the findings. Notwithstanding the cohorts, women demonstrated a good degree of HIV/AIDS knowledge; however, the knowledge about other STIs/STDs was not very reassuring.

Relative to female sex workers (FSWs), transgender women appear to have less KAP and awareness of pre-exposure prophylaxis (PrEP) [26,42,43]; despite PrEP’s critical role in HIV prevention, especially for high-risk populations. Transgender women in Asia face discrimination and violence in healthcare settings, which is rooted in the region’s profoundly traditional and patriarchal culture, which accentuates male dominance [26,43,44]. This cultural bias, which stems from the assumption that transgender women deviate from male gender norms, perpetuates stigmatization, obstructing transgender women’s access to vital HIV care and perpetuating the cycle of infection [45]. Moreover, the lack of gender-affirming healthcare expertise exacerbates the problem, erecting barriers to sufficient treatment and exacerbating their precariousness [44,46]. Consequently, many transgender women are denied the necessary medical care and HIV prevention information for effective HIV prevention. In response, PrEP emerges as a pivotal tool, providing a discrete, self-empowered prevention option that, when integrated with comprehensive strategies encompassing testing, behavioural interventions, and community engagement, has the potential to mitigate the elevated HIV susceptibility that transgender women frequently face.

When women’s KAP levels concerning HIV/STIs/STDs are evaluated, and they are considered in conjunction with the power dynamics that surround them, it becomes very evident that they are at great risk. Although most married women in Asia understood STI/STD, most were unaware of genital discharge [4] and STI/STD prevention techniques [37]. In South Asia, child marriage is prevalent, causing young girls a greater risk of abnormal vaginal discharge [4]. Young girls are forced to have sex with spouses who are often considerably older and have a history of several sexual partners, which results in the spread of STIs/STDs) [4,25]. Additionally, women’s ability to arrange safe sexual encounters with their partners may be hindered by the power imbalances that exist within their relationships. Men tend to play more dominant roles in sexual interactions in most Asian cultures, which includes making decisions about when and how sexual activities will take place, as well as whether condoms will be used [4,25,37]. Married men who engage in risky sexual behaviour with female sex workers [20,47] and transgender women [26,42] also put their spouses in jeopardy of contracting STIs/STDs or HIV as a result of their extramarital affairs or high-risk sexual behaviours outside the marriage. In a single study on unmarried women in Lebanon [7], they were aware of the risk of STI/STD and undesired pregnancy but knew little about contraception, which could lead to the likelihood of unsafe abortions. Nations with prevalent religious practices often share a common pattern, wherein children are educated from an early age to practice abstinence, resulting in limited dissemination of contraceptive and STI/STD knowledge) [7,23,25]. 

In the sexual entertainment industry in Asia, it is a common practice for female employees to refer to their male sexual partners as “lovers” [20,47,48]. This mentality contributes to the non-use or inconsistent use of condoms as a demonstration of mutual trust between the couple, and it shows the need for a tailored approach for policy interventions amongst female sex workers. 

The prevalence of mother-to-child (MTCT) HIV transmission in Asia is quite low compared to other regions, and mother-to-child HIV transmission has been successfully addressed through education programmes [28,29,49]. However, mothers were not aware that HIV can also be transmitted via breastfeeding [8,29]. It is critical to provide prevention of mother to child transmission (PMTC) services in order to eradicate the possibility of MTCT and achieve the goal of having no new HIV infections among children. PTMCT interventions are vital for pregnant women to have in order for them to be able to make an informed decision regarding childrearing, nursing, and childbirth (caesarean section) [28,29]. 

A study conducted by [19] revealed that adolescent girls who engaged in sexual and reproductive discussions with their mothers exhibited a favourable disposition toward safe sexual practices and menstruation. Conversely, Ref. [41] determined that residents of women’s shelter homes perceived premarital sexual activity as morally unacceptable. Misconceptions regarding premarital sex have the potential to engender stigmatization and discriminatory attitudes toward individuals who engage in sexual activity prior to marriage. This stigmatization can effectively impede candid dialogues pertaining to sexual health, constrain access to pertinent information, and dissuade women from seeking appropriate care and support. Some papers related attitudes and perceptions within the same definition, where both attitude and perception were closely related psychological constructs [27]. Consequently, in a study carried out by [35], it was discerned that women inhabiting urban locales demonstrated greater compassion toward fellow women afflicted by HIV, in contrast to their rural counterparts.

### 3.3. Knowledge, Awareness, and Perception (KAP) about HIV, STI/STD Based on Sociodemographic Profiles

RQ3 focused on identifying susceptible groups among women who lack KAP of HIV and STI/STD. Table 3 summarises the sociodemographic profiles influencing women’s levels of KAP. Specifically, it includes studies that demonstrated at least one statistically significant outcome (*p* < 0.05) related to knowledge, awareness, or perception. This classification of high or low was based on the individual studies.

Education and socioeconomic status are positively associated with KAP [22,37], but other factors like cultural and social norms [7], access to healthcare [33], and stigma and discrimination [44,46] can also affect it. Ref. [26] discovered that trans women with a high school diploma were more likely to use pre-exposure prophylaxis. Demographic profiles with women earners, whether from sex work or other industries, were more likely to have higher KAP [23,29,31] than those from lower income families [27,37]. 

Increased phone usage and media exposure can influence the KAP of women in Asia [4,23,25,31,32,33,40,52]. This is particularly evident amongst younger females in Asia [4,32,33]. These findings emphasise the necessity of using mobile phones and mass media to disseminate health-related information to women in Asia.

Religious perspectives regarding sexuality and birth control can influence the utilization of contraceptives among women. In specific societal contexts, contraceptive practices can be at odds with religious doctrines that emphasise procreation and larger family sizes) [7,36]. Consequently, women residing in such communities might exhibit limited knowledge, attitudes, and perception (KAP) related to contraception, often encountering societal pressures to abstain from its use. However, it is noteworthy that some religious teachings in Asia advocate for responsible family planning and endorse the adoption of contraceptive methods [7,50].

Married women in Asia may have better awareness of STI/STD [23,50]. In many Asian civilizations, marriage is viewed as a socially acceptable type of sexual conduct [53]. Hence, married women may have been exposed to greater knowledge about sexual health and the dangers of STIs/STDs through premarital counselling and educational intervention programmes [23]. In many traditional Asian communities, women are required to prioritise the welfare of their family over their own [30]. This involves preventing the transmission of STIs/STDs to partners and children. Hence, married women may be more motivated to learn about STI/STD prevention since they may feel a higher sense of responsibility for their family’s health [24,50], thus creating higher awareness compared to single unmarried women [7]. 

### 3.4. Risky Behaviors and HIV/STI/STD among Women in Asia 

This final section will focus on RQ4, which investigated the forms of risky behaviours that women in Asia indulge in due to a lack of KAP and that lead to an increased risk of contracting STI/STD. Table 4 summarises the findings for this section. 

The absence of protection during intercourse (*n* = 17) is the most dangerous behaviour identified in this analysis, with female sex workers and transgender women being the most susceptible. In Asia, female sex workers and transgender women may value monetary profit over their own safety and security [54,58,71]. This may cause them to engage in unprotected intercourse with higher-paying clients. Furthermore, having several sexual partners is an employment hazard, especially for women who engage in transactional sex [52,65,69]. In addition, stigma and prejudice against female sex workers in Asia might make it difficult for them to discuss condom use with customers or partners out of fear of losing clients or being subjected to violence [58]. Ref. [47] found females who started exchanging sex for money at a younger age had a higher chance of contracting STIs/STDs compared to older women. This is due to the lack of awareness amongst them when it comes to protection and information on contraception. Furthermore, in some parts of Asia, contraception may be difficult to access [45,59,75], expensive [39], and in some cases prove to be a taboo [7]. This can make it challenging for women to use protection consistently.

Patriarchy is deeply rooted in Asian countries; hence, married women in this region may experience power asymmetries in sexual relationships, where they cannot negotiate condom use or dictate the conditions of sexual activity [4,25,37]. Similarly, migrant wives (wives of husbands who leave their home country to find employment elsewhere) face a greater risk of STI/STD transmission due to their husbands’ sexual exploits [50,66].

Substance use, such as alcohol [61] or drugs [73], can impair judgment and increase the likelihood of engaging in risky sexual behaviours. Ref. [72] found women who injected drugs had decreased inhibitions and increased sexual drive resulting in an increased probability of engaging in risky sexual behaviour, including indulging in unprotected sex with multiple partners.

## 4. Conclusions

Based on research published over the preceding five years, this scoping review examined the variety of studies undertaken on the knowledge, awareness, perception (KAP) and risky behaviours of Asian women in connection to sexually transmitted infections and diseases (STIs/STDs). The vast bulk of the research was conducted in Southeast Asia, focusing on several cohorts, including high-risk populations (including pregnant mothers, FSWs, and transgender women, among others), adolescents, and women. Most studies indicated low levels of knowledge, awareness, and perception, and this pattern was shown to be more pronounced among individuals with low levels of education, low socioeconomic status, rural residence, and/or sex or industrial employment. Owing to a low condom-use negotiation capacity (aggravated by alcohol and substance abuse), lack of awareness and education, etc., it was discovered that high-risk people participate in unsafe sex most of the time, including having several partners. This was demonstrated to be true. To prevent the spread of sexually transmitted infections and diseases (STIs/STDs) among women in Asia, greater time, effort, and resources are required, according to the findings of this review. This includes improving healthcare education in schools, households, and communities.

This study contributes important knowledge to the literature; however, it does come with limitations. First, the articles were limited to those available in the four electronic databases and focusing on a single cohort, which was women in the reproductive age brackets and transgender women. All the studies assessed KAP through self-reported questionnaires, and thus may have been affected by bias leading to an overestimation/underestimation of the levels of KAP. Therefore, the results should be interpreted with caution. Second, a vast majority of the studies were from Southeast Asia, probably contributing to the high heterogeneity in the results. This could also be attributed to the disproportionate sample populations investigated, instruments with varying cut-off points, etc. Third, the scarcity of Chinese studies identified could be attributed to the possibility that such studies were not available in the databases examined in the course of this paper’s research. Chinese research papers are often published in Chinese-language journals and only accessible via SinoMed and the Chinese Social Sciences Citation Index, making them less accessible in English-language databases.

## Figures and Tables

**Table 1:** Descriptive statistics.

Study Characteristics	Frequency (*n*)	Percentage (%)
*Year of publication*		
2018	15	22.1
2019	15	22.1
2020	17	25.0
2021	17	25.0
2022	4	5.9
*Region*		
East Asia (China, including Hong Kong)	11	16.2
South Asia (India, Sri Lanka, Nepal, Bangladesh, Pakistan)	20	29.4
Southeast Asia (Indonesia, Thailand, Malaysia, Vietnam, Cambodia)	24	35.3
Southwest Asia (Iran, Lebanon)	12	17.6
Multiple	1	1.5
*Study Design*		
Cross-sectional	52	76.5
Mixed methods	2	2.9
Specific cohort (brothel madams)	1	1.5
Others *	13	19.1
*Focus* *		
Sexual/Risky behaviour	31	45.6
Awareness	7	10.3
Knowledge	25	36.8
Perception	5	7.4
*Aspect* *		
HIV	54	79.4
STD/STI	13	19.1
*Cohort*		
Female sex workers	19	27.9
Transgender women	15	22.1
Married/Pregnant women	12	17.6
Young/Adolescent girls	4	5.9
Women of various age groups	10	6.8
Others *	8	11.8
*Scale Used*		
Self-administered/Own survey	21	30.9
Integrated behavioural and biological survey	3	4.4
Demographic health survey	4	5.9
Questionnaires (based on previous literature, national standards, etc.)	24	35.3
Others *	16	23.5
*Sociodemographic* *		
Age	14	20.6
Education	22	32.4
Marital status	11	16.2
Socioeconomic/income	15	22.1
Occupation	7	10.3
Religion/ethnicity	3	4.4
Place of birth	2	2.9
Others (sexual orientation, sexual habits, cultural factors, etc.)	8	11.8

Note: *—Others (study design) refers to cohort studies, observational studies, case control, etc. (full list available in Supplementary Materials); Others (cohort) refers to prisoners/inmates, refugee women, mother–daughter pairs, women persons who inject drugs (PWID), female patients, homeless women, etc.; Others (scale used) refers to blood samples, ELISA, face-to-face interviews, etc.; Focus, Aspect, and Sociodemographic numbers do not sum up to 70 due to some studies being multiple-focus, -aspect, and -sociodemographic.

**Table 2:** Levels of KAP about HIV, STI/STD among women in Asia.

Topic	Study	Cohort, Size (*n*)	* Knowledge		Awareness		Positive Attitude/Perception	
			Level (%)	Scale (Mean)	Level (%)	Scale	Level (%)	Scale
HIV/ AIDS	[21]	Girls Size = 188	70.7%					
	[22]	TW Size = 360			17.1%			
	[23]	Young women Size = 600	67.3%				77%	
	[24]	Women Size = 45,067	53.6%					
	[25]	Married women Size: 12,593	62%					
	[26]	TW Size = 361			20.2%			
	[27]	Married women Size: 13,558	28%				55%	
	[28]	Pregnant women Size: 350	14.3%				84%	
	[29]	Pregnant women Size: 200	82.5%					
	[30]	Housewives Size: 32	16%					
	[31]	FSW Size: 90	81%					
	[32]	Women aged 15–49 Size: 9252	42.4%					
	[8]	Pregnant women Size: 400			74%			
	[33]	Women aged 15–49 Size: 25,895	88.74%				39.72%	
	[34]	TW Size: 222			33.3%		49.1%	
	[35]	Women Size: 44,921	19.1%				15.4%	
STI/STD	[36]	Women aged 15–45 Size: 241				13.96 ± 8.7		
	[7]	Unmarried women Size: 491	8.8%					
	[4]	Married women Size: 41,777	71%		14%			
	[37]	Married women Size: 12,364	34.8%		34%			
	[38]	Married women Size: 84			61.9%			
	[5]	FSW Size: 173		62.1				
	[19]	Mother–daughter pairs Size: 112					48.2% (adolescent girls) 41.0% (mothers)	
	[39]	TW Size = 127	33.9%					
	[40]	Adolescent girls (age 16–18) Size: 792	54.8%					
	[41]	Inmates of women’s shelter homes Size: 60	33.3%	10.9/15				23.1/25

FSW = female sex workers/commercial sex workers; TW = trans women/transgender women. *—Indicates possession of knowledge based on population.

**Table 3:** Levels of KAP based on sociodemographic profiles of women in Asia.

Sociodemographic Profiles		Levels of Knowledge, Awareness, or Perception	
		High	Low
		Studies	
Education level	High	[32]	[7]
	Low	[21]	[22,27,30,37,45,50]
Age	>30	[24,51]	[22,45,50]
	<30	[4,23,25,31,32,33,40,52]	[7,28,35,37,41]
Marital status	Married	[4,23,24,25,29,37,50]	[28,30]
	Unmarried/Single	[32,33]	[7]
Socioeconomic status	High	[40]	[35]
	Low	[25]	[22,41,50]
Geographical location	Rural	[4,25]	[37]
	Urban	[24]	[22,28,35]
Religion	Identified with a religion	[23,25,40]	[7,28,36]
Occupation	Sex/commercial workers	[29,31,52]	[22,45]
	Employed	[23,33,51]	[30,50]
	Unemployed	[4]	[27,37]

Note: Only sociodemographic profiles with more than two studies are highlighted; significant results only; age capped at 30 by calculating median age of all studies.

**Table 4:** HIV/STI/STD risky behaviours among women.

Type	Risky Behaviour	Cohort	Studies
Sexual	No/infrequent use of protective and/or preventive measures	Migrant workers	[50]
		Transgender	[39,54,55,56,57]
		FSW */inmates	[42,47,52,58,59,60,61,62,63]
		Homeless community	[36]
		Women	[64]
	Multiple sex partners	Transgender	[39,44,57]
		FSW *	[42,59,65]
		Married community	[66]
		Women	[67,68]
Social	Fear of rejection, discrimination, and stigmatization	FSW *	[58]
		Migrant workers	[50]
	Lacking awareness and knowledge	Transgender	[22,39]
		FSW *	[52,60,69]
		Adolescents	[24]
		Married community	[24,37]
		Homeless community	[36]
		Women (married/pregnant)	[25,27,28,29,67]
Personal	Personality behaviour: impulsivity	FSW */inmates	[6,51]
	Occupation-related risk	FSW *	[48,52,70,71]
	Marital status	FSW *	[63,70]
		Married women	[4]
	Education	PWID *	[72,73]
		FSW *	[52]
		Young women	[23]
		Single, unmarried women	[7]
	Alcohol consumption	FSW *	[61]
	Others Inject drugs, sexually active, partner/childhood sex abuse, difficulty in condom procurement, substance/alcohol abuse, sharing needles	FSW *	[70]
		Transgender	[44,55]
		Women	[68,74]
		PWID *	[72,73]

* Note: FSW = female/commercial sex worker; PWID = person who injects drugs.

## Supplementary Materials

The following supporting information can be downloaded at: https://www.mdpi.com/article/10.3390/healthcare11192643/s1.

## References

[1] Stöver H., Michels I.I. (2022). Development of social work in prevention and treatment of HIV/AIDS and HCV in Central Asia. Drugs Habits Soc. Policy.

[2] Fongkaew K., de Lind van Wijngaarden J.W., Tepjan S., Chonwanarat N., Akkakanjanasupar P., Newman P.A. (2022). ‘No test, no disease’: Multilevel barriers to HIV testing among young men who have sex with men and transgender women in three semi-urban areas in Thailand. Cult. Health Sex..

[3] Sukartini T., Nursalam N., Arifin H. (2021). The determinants of willingness to care for people living with HIV-AIDS: A cross-sectional study in Indonesia. Health Soc. Care Community.

[4] Huda M., Ahmed M.U., Uddin M., Hasan M.K., Uddin J., Dune T.M. (2022). Prevalence and demographic, socioeconomic, and behavioral risk factors of self-reported symptoms of sexually transmitted infections (STIs) among ever-married women: Evidence from Nationally representative surveys in Bangladesh. Int. J. Environ. Res. Public Health.

[5] Miankouhi T.A., Malakouti J., Mirghafourvand M., Farshbaf-Khalili A. (2018). Knowledge regarding sexually transmitted infections and socio-demographic predictors in women with high-risk sexual behaviors. Arch. Clin. Infect. Dis..

[6] Arends R.M., Nelwan E.J., Soediro R., van Crevel R., Alisjahbana B., Pohan H.T., von Borries AK L., Schene A.H., van der Ven A.J., Schellekens A.F. (2019). Associations between impulsivity, risk behavior and HIV, HBV, HCV and syphilis seroprevalence among female prisoners in Indonesia: A cross-sectional study. PLoS ONE.

[7] Hamdanieh M., Ftouni L., Al Jardali B.A., Ftouni R., Rawas C., Ghotmi M., El Zein M.H., Ghazi S., Malas S. (2021). Assessment of sexual and reproductive health knowledge and awareness among single unmarried women living in Lebanon: A cross-sectional study. Reprod. Health.

[8] Upadhyay M., Lata K., Yadav T.C., Mahendru R., Siwach S., Lakra P. (2021). Knowledge, Attitude and Perception of HIV/Aids Among Antenatal Women and its Correlation with their Socio-Demographic Profile: Study from a Tertiary Care Centre of Northern India. J. Obstet. Gynecol. India.

[9] Fauk N.K., Mwanri L., Hawke K., Mohammadi L., Ward P.R. (2022). Psychological and social impact of HIV on women living with HIV and their families in low-and middle-income Asian countries: A systematic search and critical review. Int. J. Environ. Res. Public Health.

[10] Omidi T., Oshnouei S., Mahdi-Akhgar M., Mohammadian-Khoshnoud M., Mohammadi Y. (2023). Barriers to Condom Use among Female Sex Workers: A Systematic Review and Meta-Analysis. Curr. Womens Health Rev..

[11] Tavakoli F., Khezri M., Tam M., Bazrafshan A., Sharifi H., Shokoohi M. (2021). Injection and non-injection drug use among female sex workers in Iran: A systematic review and meta-analysis. Drug Alcohol Depend..

[12] Teng F., Sha Y., Fletcher L.M., Welsch M., Burns P., Tang W. (2023). Barriers to uptake of PrEP across the continuum among transgender women: A global scoping review. Int. J. STD AIDS.

[13] Bhagavathula A.S., Clark C.C., Sharma R., Chhabra M., Vidyasagar K., Chattu V.K. (2021). Knowledge and attitude towards HIV/AIDS in India: A systematic review and meta-analysis of 47 studies from 2010–2020. Health Promot. Perspect..

[14] Patsani P., Parida J., Panda A., Jena S., Behera S.S., Pradhan A., Patra P.K., Pati S., Kaur H., Acharya S.K. (2023). Knowledge, beliefs and practices towards HIV/AIDS among adolescents in India: A scoping review protocol. PLoS ONE.

[15] Bogale A.L., Teklehaymanot T., Haidar Ali J., Kassie G.M. (2021). Knowledge, attitude and practice of cervical cancer screening among women infected with HIV in Africa: Systematic review and meta-analysis. PLoS ONE.

[16] Balakrishnan V., Yong K.K., Tiong C.K., Ng N.J.S., Ni Z. (2023). A Scoping Review of Knowledge, Awareness, Perceptions, Attitudes, and Risky Behaviors of Sexually Transmitted Infections in Southeast Asia. Healthcare.

[17] Arksey H., O’Malley L. (2005). Scoping studies: Towards a methodological framework. Int. J. Soc. Res. Methodol..

[18] Tricco A.C., Lillie E., Zarin W., O’Brien K.K., Colquhoun H., Levac D., Moher D., Peters M.D., Horsley T., Weeks L. (2018). PRISMA extension for scoping reviews (PRISMA-ScR): Checklist and explanation. Ann. Intern. Med..

[19] Noe MT N., Saw Y.M., Soe P.P., Khaing M., Saw T.N., Hamajima N., Win H.H. (2018). Barriers between mothers and their adolescent daughters with regards to sexual and reproductive health communication in Taunggyi Township, Myanmar: What factors play important roles?. PLoS ONE.

[20] Wilson A., Wang Y.Y., Chen R., Cen P., Wang Y., Yao X., Wang T., Li S., Yan H. (2021). A thematic analysis of experiences of HIV risks among female sex workers in the Yunnan-Vietnam Chinese border region. BMC Womens Health.

[21] Jozani Z.B., Badie B.M., Bayanolhagh S., Mohammadifirouzeh M., Ahsani-Nasab S., Vakili F., Seyedalinaghi S., Golchehregan H., Foroughi M., Mohraz M. (2019). Knowledge, Attitude and Practice Towards HIV/AIDS Conjoint with HIV, HBV, HCV and HSV2 Serosurveys among Girls from Dysfunctional Families in Tehran, Iran. J. Int. Transl. Med..

[22] Chakrapani V., Shaikh S., Arumugam V., Chawla U., Mehta S. (2021). Factors Influencing Willingness to Use Human Immunodeficiency Virus Preexposure Prophylaxis Among Transgender Women in India. Transgender Health.

[23] Devarayasamudram S., De Gagne J.C., Kurudi N.P., Kang H.S. (2018). Effectiveness of a Structured teaching program on knowledge and attitudes toward hiv among young women in india. J. Community Health Nurs..

[24] Efendi F., Pratama E.R., Hadisuyatmana S., Indarwati R., Lindayani L., Bushy A. (2020). HIV-related knowledge level among Indonesian women between 15 years and 49 years of age. Afr. Health Sci..

[25] Haque M.A., Hossain MS N., Chowdhury MA B., Uddin M.J. (2018). Factors associated with knowledge and awareness of HIV/AIDS among married women in Bangladesh: Evidence from a nationally representative survey. SAHARA-J J. Soc. Asp. HIV/AIDS.

[26] Galka J.M., Wang M., Azwa I., Gibson B., Lim S.H., Shrestha R., Wickersham J.A. (2020). Willingness to use pre-exposure prophylaxis (PrEP) for HIV prevention and PrEP implementation preferences among transgender women in Malaysia. Transgender Health.

[27] Iqbal S., Maqsood S., Zafar A., Zakar R., Zakar M.Z., Fischer F. (2019). Determinants of overall knowledge of and attitudes towards HIV/AIDS transmission among ever-married women in Pakistan: Evidence from the Demographic and Health Survey 2012–2013. BMC Public Health.

[28] Irfan A., Kazmi S.K., Anwar Z., Khan F.M.A., Khan J., Arif Y., Noor M., Shakil A., Hassan W., Ali R. (2019). Knowledge and attitude of pregnant women regarding HIV transmission, prevention and associated factors in Karachi, Pakistan—A cross-sectional study. Sex. Reprod. Healthc..

[29] Jahangir Y.T., Arora A., Liamputtong P., Nabi M.H., Meyer S.B. (2020). Provider perspectives on sexual health services used by Bangladeshi women with mHealth digital approach: A qualitative study. Int. J. Environ. Res. Public Health.

[30] Kurniawati H.F., Kurniawati H.F. (2021). Characteristics, Source of Information and Knowledge of Housewives about Transmission of HIV and Aids. Pak. J. Med. Health Sci..

[31] Sinha A., Goswami D.N., Haldar D., Choudhury K.B., Saha M.K., Dutta S. (2020). Sociobehavioural matrix and knowledge, attitude and practises regarding HIV/AIDS among female sex workers in an international border area of West Bengal, India. J. Fam. Med. Prim. Care.

[32] Son N.V., Luan H.D., Tuan H.X., Cuong L.M., Duong NT T., Kien V.D. (2020). Trends and factors associated with comprehensive knowledge about HIV among women in Vietnam. Trop. Med. Infect. Dis..

[33] Virdausi F.D., Efendi F., Kusumaningrum T., Adnani Q.E.S., McKenna L., Ramadhan K., Susanti I.A. (2022). Socio-Economic and Demographic Factors Associated with Knowledge and Attitude of HIV/AIDS among Women Aged 15–49 Years Old in Indonesia. Healthcare.

[34] Yan L., Yan Z., Wilson E., Arayasirikul S., Lin J., Yan H., McFarland W. (2021). Awareness and willingness to use HIV pre-exposure prophylaxis (PrEP) among trans women in China: A community-based survey. AIDS Behav..

[35] Zarei E., Khabiri R., Tajvar M., Nosratnejad S. (2018). Knowledge of and attitudes toward HIV/AIDS among Iranian women. Epidemiol. Health.

[36] Allahqoli L., Fallahi A., Rahmani A., Higgs P. (2018). The prevalence of human immunodeficiency virus infection and the perceptions of sexually transmitted infections among homeless women. Nurs. Midwifery Stud..

[37] Maqsood S., Iqbal S., Zakar R., Zakar M.Z., Fischer F. (2021). Determinants of overall knowledge and health behaviours in relation to hepatitis B and C among ever-married women in Pakistan: Evidence based on Demographic and Health Survey 2017–2018. BMC Public Health.

[38] Mariani A., Seweng A., Ruseng S.S., Moedjiono A.I., Abdullah T., Anshary A., Nur R., Basir M. (2021). The relationship between knowledge and personal hygiene and the occurrence of sexually transmitted diseases at the Community Health Center Talise, Palu. Gac. Sanit..

[39] Nematollahi A., Gharibzadeh S., Damghanian M., Gholamzadeh S., Farnam F. (2022). Sexual behaviors and vulnerability to sexually transmitted infections among transgender women in Iran. BMC Womens Health.

[40] Zakaria M., Karim F., Mazumder S., Cheng F., Xu J. (2020). Knowledge on, attitude towards, and practice of sexual and reproductive health among older adolescent girls in Bangladesh: An institution-based cross-sectional study. Int. J. Environ. Res. Public Health.

[41] Zin N.M., Ishak I., Manoharan K. (2019). Knowledge, attitude and practice towards sexually transmitted diseases amongst the inmates of women shelters homes at Klang Valley. BMC Public Health.

[42] Jommaroeng R., Richter K.A., Chamratrithirong A., Soonthorndhada A. (2020). The effectiveness of national HIV prevention education program on behavioral changes for men who have sex with men and transgender women in Thailand. J. Health Res..

[43] Rutledge R., Morozova O., Gibson B.A., Altice F.L., Kamarulzaman A., Wickersham J.A. (2018). Correlates of recent HIV testing among transgender women in Greater Kuala Lumpur, Malaysia. LGBT Health.

[44] Shan D., Yu M.-H., Yang J., Zhuang M.-H., Ning Z., Liu H., Liu L., Han M.-J., Zhang D.-P. (2018). Correlates of HIV infection among transgender women in two Chinese cities. Infect. Dis. Poverty.

[45] Cempaka R., Wardhani B., Sawitri A.A.S., Januraga P.P., Bavinton B. (2020). PrEP use awareness and interest cascade among msm and transgender women living in Bali, Indonesia. Trop. Med. Infect. Dis..

[46] Shrestha R., Galka J.M., Azwa I., Lim S.H., Guadamuz T.E., Altice F.L., Wickersham J.A. (2020). Willingness to use HIV self-testing and associated factors among transgender women in Malaysia. Transgender Health.

[47] Abadi M.-A., Abolghasemi J., Rimaz S., Majdzadeh R., Shokoohi M., Rostami-Maskopaee F., Merghati-Khoei E. (2018). High-risk behaviors among regular and casual female sexworkers in Iran: A report fromwestern Asia. Iran. J. Psychiatry Behav. Sci..

[48] Tuot S., Teo A.K.J., Chhoun P., Mun P., Prem K., Yi S. (2020). Risk factors of HIV infection among female entertainment workers in Cambodia: Findings of a national survey. PLoS ONE.

[49] Khetsuriani N., Lesi O., Desai S., Armstrong P.A., Tohme R.A. (2022). Progress Toward the Elimination of Mother-to-Child Transmission of Hepatitis B Virus—Worldwide, 2016–2021. Morb. Mortal. Wkly. Rep..

[50] Ayuttacorn A., Tangmunkongvorakul A., Musumari P.M., Srithanaviboonchai K., Jirattikorn A., Aurpibul L. (2019). Disclosure of HIV status among Shan female migrant workers living with HIV in Northern Thailand: A qualitative study. PLoS ONE.

[51] Wu P., Dong W.M., Rou K., Dong W., Zhou C., Chen X., Zheng J., Scott S.R., Wu Z. (2019). HIV-positive clients of female sex workers in Hunan Province, China: A mixed methods study assessing sexual relationships and risk behavior by type of partner. BMC Public Health.

[52] Yu J., Nehl E.J., Dinh V.P., Liang B., Son N.V., Meng D., Zhang Y., Jiang J., Huang J., Ning C. (2020). Vietnamese female sex workers in rural cross-border areas of Guangxi, China: Migration and HIV/STI risk behaviors. AIDS Care.

[53] Song C., Xie H., Zhou Y., Chatterjee J.S. (2023). Sex life and sexuality among tongqi: Doing gender and heterosexuality. Cult. Health Sex..

[54] Khalid H., Martin E.G. (2019). Relationship between network operators and risky sex behaviors among female versus transgender commercial sex workers in Pakistan. AIDS Care.

[55] Shan D., Ning Z., Yu M., Zheng H., Yang J., Gong H., Li J., Liu H., Liu L., Wang V. (2022). HIV incidence and risk factors among transgender women and cisgender men who have sex with men in two cities of China: A prospective cohort study. Infect. Dis. Poverty.

[56] Storm M., Deuba K., Damas J., Shrestha U., Rawal B., Bhattarai R., Marrone G. (2020). Prevalence of HIV, syphilis, and assessment of the social and structural determinants of sexual risk behaviour and health service utilisation among MSM and transgender women in Terai highway districts of Nepal: Findings based on an integrated biological and behavioural surveillance survey using respondent driven sampling. BMC Infect. Dis..

[57] Wansom T., Muangnoicharoen S., Nitayaphan S., Kitsiripornchai S., Crowell T.A., Francisco L., Gilbert P., Rwakasyaguri D., Dhitavat J., Li Q. (2021). Risk Factors for HIV sero-conversion in a high incidence cohort of men who have sex with men and transgender women in Bangkok, Thailand. Eclinicalmedicine.

[58] Emmanuel F., Achakzai B.K., Reza T. (2021). Prevalence and factors associated with HIV epidemic among female sex workers in Pakistan: Results of the fifth round of integrated biological and behavioural surveillance. Sex. Transm. Infect..

[59] Jiang T., Pan X., Ma Q., Jiang J., Chen L., Wang H., Zhou X., Chen W. (2021). Characteristics of low-tier female sex workers who engage in commercial sex with old male clients in Zhejiang province, China: A cross-sectional study. BMJ Open.

[60] Kakchapati S., Gautam N., Kc K.P., Rawal B.B. (2018). HIV awareness and safe sexual behaviors among female sex workers in Kathmandu valley of Nepal. HIV/AIDS-Res. Palliat. Care.

[61] Khumaidi, Yona S., Waluyo A. (2021). Condom-use negotiation, alcohol consumption, and HIV-risk sexual behavior among female sex workers in Kupang, East Nusa Tenggara, Indonesia: A cross-sectional study. J. Public Health Res..

[62] Mohammadi Gharehghani M.A., Khosravi B., Irandoost S.F., Soofizad G., Lebni J.Y. (2020). Barriers to condom use among female sex workers in Tehran, Iran: A qualitative study. Int. J. Womens Health.

[63] Zhou X., Ma Q., Pan X., Chen L., Wang H., Jiang T. (2020). The prevalence and correlates of oral sex among low-tier female sex workers in Zhejiang province, China. PLoS ONE.

[64] Khuat T.H., Do T.T., Nguyen V.A.T., Vu X.T., Nguyen P.T.T., Tran K., Ho M.T., Nguyen HK T., Vuong T.T., La V.P. (2018). The dark side of female HIV patient care: Sexual and reproductive health risks in pre-and post-clinical treatments. J. Clin. Med..

[65] Guida J., Hu L., Liu H. (2019). Sexual behavior with noncommercial partners: A concurrent partnership study among middle-aged female sex Workers in China. J. Sex Res..

[66] Ranjan A., Kumar P., Ahmad S., Pandey S., Detel R. (2019). Pattern of sexual behavior among people in a rural area of Bihar: A qualitative study on wives of migrant workers. J. Fam. Med. Prim. Care.

[67] Pei R., Ji-Ke C., Yu G., Yang Y., Nan L., Liao Q., Wang J., Liu D., Yang S. (2020). Sexual behaviors related to HIV infection in Yi women of childbearing age in rural areas of southwest China. AIDS Care.

[68] Samal N., Padhi S., Burman L. (2019). Seroprevalence of hepatitis B infection among pregnant women in Southern Odisha. Indian J. Med. Spec..

[69] Manathunge A., Barbaric J., Mestrovic T., Beneragama S., Bozicevic I. (2020). HIV prevalence, sexual risk behaviours and HIV testing among female sex workers in three cities in Sri Lanka: Findings from respondent-driven sampling surveys. PLoS ONE.

[70] Biswas S., Sinha A., Rajan S., Khan P.K., Joshi D.S., Saha M.K. (2020). Human immunodeficiency virus prevalence and high-risk behavior of home-based and nonhome-based female sex workers in three high-prevalent North-Eastern States of India. Indian J. Public Health.

[71] Hu L., Wu G., Lu R., Zhu H., Qiu H., Jing D., Ye M. (2020). Changing trends of HIV, syphilis, HCV infections and behavioural factors among female sex workers in Chongqing, China: Findings from six serial surveillance surveys. BMJ Open.

[72] Damas J., Storm M., Pandey L.R., Marrone G., Deuba K. (2021). Prevalence of HIV, Hepatitis C and its related risk behaviours among women who inject drugs in the Kathmandu Valley, Nepal: A cross-sectional study. Ther. Adv. Infect. Dis..

[73] Stoicescu C., Cluver L.D., Spreckelsen T., Casale M., Sudewo A.G. (2018). Intimate partner violence and HIV sexual risk behaviour among women who inject drugs in Indonesia: A respondent-driven sampling study. AIDS Behav..

[74] Hamzeh B., Moradi Z., Najafi F., Moradinazar M. (2019). Pattern of substance abuse and prevalence of risk factors of HIV and hepatitis among addicted women in western Iran. Int. J. Prev. Med..

[75] Bavinton B.R., Mahendra IG A.A., Kaldor J., Law M., Grulich A.E., Januraga P.P. (2021). Estimation of potential HIV transmission risk in recent anal intercourse events among men who have sex with men and transgender women in Bali, Indonesia. Trop. Med. Infect. Dis..

