# Supplementary material for: Understanding Women’s Knowledge, Awareness, and Perceptions of STIs/STDs in Asia: A Scoping Review
Source: Healthcare (Basel). 2023 Sep 28;11(19):2643. doi: 10.3390/healthcare11192643 (PMC10572356; doi:10.3390/healthcare11192643)
Supplement: Supplementary file 1 [file healthcare-11-02643-s001.zip › Supplementary Table 1.pdf]

No	Author	Year	Country	Aim	Cohort / Size	Type of Disease/ Aspect	Focus	Scale	Methodology	Study Design	Key Results
1	Abadi et al.	2018	Iran	This study aims at comparing the high risk behaviours between regular and casual sex workers in Iran , Western Asia where female sex workers are at risk of obtaining STI, especially HIVs	Cohort : Female Sex workers<br>Size : 184	HIV	Risk behaviours	Standard questionnaire	Survey	Cross-sectional questionnaire based study	FSW with very low educational level : >33.3%<br>FSW with higher educational level : 33.3%<br><br>Agree to take HIV testing (72.7%) : 57.9% (Casual) , 42.1% (Regular)<br>Disagree to take HIV testing (27.3%) : 44% (Casual) , 56% (Regular)<br><br>Condom use (latest year) : 42.3% were consistent , 57.7% were inconsistent<br><br>Drug used before sex (past year) : 38.4% were consistent , 61.6% were inconsistent
2	Allahqoli Et Al	2018	Tehran , Iran	The aim of the present study was to assess the prevalence of HIV infection and the perceptions of STIs among homeless women in Tehran, Iran.	Cohort :Women who were Iranians, aged 15–45, had been sexually active in the last 12 months and were able to understand and speak Persian, in drop-in centers and night shelters in Tehran, Iran<br>Size : 241	STD	Perception of STI among homeless woman	Self-administered questionnaire	Survey	Cross-sectional study	Around 82.6% of participants perceived that they were at low risk for developing STIs, while only 8.3% of them (two cases) perceived high STI risk. Results also indicated that HIV positivity had significant relationships with overall STI risk perception and its insufficient knowledge and inconsistence condom use subscales.
3	Arends Et Al	2019	Indonesia	This cross-sectional study aims to delineate the contributions of different aspects of impulsivity to risk behavior, among female inmates living in a prison in Jakarta	Cohort : Female prisoners<br>Size : 214	HIV , HBV , HCV , Syphilis	Risk behaviours	Self-administered questionnaire	Survey	Cross-sectional study	Motor impulsivity (BIS-motor) predicted alcohol-related risk behavior, sensitivity to reward (SPSRQ-reward) predicted drug-related risk behavior, and cognitive impulsivity (BIS-attentional) and goal-directed impulsivity (BAS-drive) predicted sexual risk behavior and being seropositive for HIV, HBV, HCV or syphilis. The associations between impulsivity and seropositivity were not mediated by risk behavior.
4	Ayuttacorn Et Al	2019	Thailand	The current study addressed this research gap in knowledge regarding HIV status disclosure and risky sexual behavior in HIV-infected female migrant workers, and focused on HIV-infected Shan female migrant workers in Northern Thailand.	Cohort : Female Migrant workers<br>Size : 18	HIV	Risky behaviours	Semi-structured questionnaire	Face to face interview	Cross-sectional study	Non-disclosure to husbands/partners was mostly related to fear of marital conflict and of losing social and financial support. Non-disclosure prevented Shan female migrant workers from negotiating condom use with their partners. Reasons for not disclosing to friends, family and other community members were mostly related to feared rejection and discrimination due to HIV-related stigma. Accounts of condomless sex in the context of HIV status disclosure suggest that gender norms and male dominance over women influenced decision-

											making for safe sex. Lastly, some female migrant workers perceived low risk of HIV transmission with good adherence to the ART.
5	Biswas et al. (2020)	2020	India	1. To describe sociodemographic and sex work charactericstics<br>2. To identify the risk factors for HIV infection with special focus on the variations between home-based (HB) and non-HB (NHB) female sex workers (FSWs) in 3 high-prevalent North-Eastern states of India: Manipur, Mizoram, and Nagaland	Cohort: FSW population from 4 districts (Aizawl, Dimapur, Imphal East, and Senapati) among 3 high HIV prevalent North-Eastern states (Manipur, Mizoram and Nagaland) in the IBBS study (National AIDS Control Organisation [NACO], 2015) during November 2014 - February 2015<br><br>Size: 1327 (435 HB; 892 NHB)	HIV	Risk factors	IBBS Questionnaire (Database from IBBS)	Face-to-face interviews	N/A (Integrated bio-behavioural surveillance?)	HIV Prevalance:<br>(NHB FSWs [7.3%] > HB FSW [4.6%])<br><br>FSW in sex work for longer duration:<br>(HB [66.7%] > NHB [60.2%])<br><br>Risk of HIV infection due to injecting drug use (NHB [11.7%] > HB [8.7%])
6	Cempaka et al.	2020	Indonesi a	To present a PrEP cascade among men who have sex with men (MSM) and transgender women (known locally as "waria") in Denpasar, Bali, from a cross-sectional survey with 220 HIV-negative MSM/waria from one clinic in Denpasar	Cohort: HIV-negative MSM and transgender women in Denpasar, Bali<br><br>Size: 220	HIV	Awareness	own survey	Survey	Cross-sectional study (**from August 2017 to April 2018)	16.4% heard of PrEP<br>2 participants had ever been on PrEP<br><br>81.4% expressed interest in using PrEP<br>78.1% were willing to do PrEP procedures
7	Chakrapan i et al	2021	India	To examine the extent of and factors influencing willingness to use PrEP among trans women in India	Cohort: Transgender women<br><br>Size: 360	HIV	Knowledge	Self-administered questionnaire	Focus group	Cross-sectional study	17.1% of participants reported having heard of PrEP before the survey<br><br>80.6% reported that they would definitely use PrEP<br><br>Trans women in sex work had 28 times higher odds of reporting willingness to use PrEP than those not in sex work
8	Damasetal 2021	2021	Nepal	In order to properly design interventions and develop programmes for women who inject drugs, this study assessed the prevalence of HIV, Hepatitis B, Hepatitis C, and syphilis and its risk behaviours among women who inject drugs	Cohort: women<br><br>Size: 160	risky behaviour	risky behaviour	modified	network sampling	cross-sectional study	The prevalence of HIV, HCV, and HBV was 8.8%, 21.3%, and 1.9%, respectively. HIV-HCV co-infection rate was 5.6%. Fifteen percent of women who inject drugs reported transactional sex for drugs or money. One in four women who inject drugs (27.5%) reported that they were imprisoned or detained for drug related reasons. In multivariable analysis, women living with HIV who inject drugs were almost four times

				in the Kathmandu Valley, Nepal.							more likely to use a previously used needle/syringe than women who inject drugs who were HIV negative, but were almost four times more likely to use a condom during sexual intercourse. Enrolment in family planning was the main determinant for using condoms in last sexual intercourse. Participants with access to HIV test and counselling (HTC) services were less likely to share needles.
9	Devarayas amudrame tal2018	2018	India	effectiveness of a structured teaching program on HIV- related knowledge and attitudes	Cohort: young women<br><br>Size: 600	HIV	Effectiveness of a Structured Teaching Program	Structured interview schedule	questionnaire	quasi-experimental pre-post	Significant differences were found in the level of the participants' knowledge of ($t = 78.89$, $p < .05$) and attitudes toward HIV after the intervention. Our findings suggest that the structured teaching program could be utilized in increasing young women's knowledge of, and improving their attitudes about, HIV.
10	Efendi et al. (2020)	2020	Indonesi a	Examine HIV-related knowledge among women in Indonesia and the associated demographic determinants that influence their access to accurate HIV-related information.	Cohort: Women between 15 and 49 years old.<br><br>Size: 45067	HIV	Knowledge	Indonesian Demographic and Health Survey 2012	Survey	Secondary analysis	More than half (53.6%) of all the women in the study had high score of HIV-related knowledge.
11	Emmanual et al. (2020)	2021	Pakistan	Determine the prevalence and factors associated with HIV infection among female sex workers (FSWs) in Pakistan.	Cohort: FSW<br><br>Size: 5660	HIV	Risky behavior	Structured questionaire based on WHO recommended guidelines	Survey, 2 rapid tests for HIV	Cross-sectional study	Weighted national HIV prevalence of 2.3% was found.<br><br>Factors independently associated with HIV in FSWs were identified.
12	Galka	2020	Malaysi a	Malaysian TW's willingness to use PrEP and their attitudes and preferences related to delivery of PrEP and PrEP-related care.	COHORT: TW<br>SIZE: 361	HIV	Attitude	own survey	online survey	in three states across west Malaysia, (Sgor, Penang & N9) + Pahang	82% of participants willing to use PrEP. Lifetime hormone use, prior postexposure pro-phylaxis use, and having completed a high school education were associated with higher willingness to use PrEP
13	Gharehgha ni	2020	Iran	identify the barriers to condom use among female sex workers in Tehran, Iran.	COHORT: FSW<br>SIZE: 22	HIV	attitude, risky behavior	own questions	Semi-structured interview	qualitative approach and conventional content analysis	Many female sex workers did not have a proper understanding of HIV and the ways of its transmission<br>Female sex workers and their clients perceived condom use as a kind of disinterest and lack of romance, so they did not use condoms in sex with sexual partners to whom they had feelings and interest.<br>Lack of motivation by sex worker, client desire to not use condom, lack of availability of condom, cultural taboo with the use of condoms among reasons condom not used
14	Guida	2019	China	investigate sexual concurrency and its associations with condom	COHORT: FSW<br>SIZE: 1245	syphilis	Risky behavior	Theory of Planned Behavior	interview	cross-sectional	Condom use was low with both commercial & non-commercial sexual partners (ie. husband or boyfriend)

				use and syphilitic infections among FSWs over 35 years old.							
15	Hamdanieh et al. (2021)	2021	Lebanon	To assess the level of sexual and reproductive health (SRH) related knowledge and awareness among single unmarried women living in Lebanon	Cohort: Single unmarried women living in Lebanon aged between 17 and 55 years<br><br>Size: n = 491	STIs	Knowledge & awareness	Self-administered questionnaire	Survey	Cross-sectional study	8.8% of all the participants had adequate knowledge. The highest level of SRH related knowledge was about pregnancy (88.0%), and the least was about contraception (13.5%).
16	Hamzeh et al. (2019)	2019	Western Iran	To investigate the pattern of substance abuse and prevalence of HIV and hepatitis risk factors among addicted women	Cohort: Addicted women referred to methadone treatment centres of Western Iran (Kermanshah province)<br><br>Size: n = 138	HIV, hepatitis	Pattern of substance abuse & risk factors	Self-administered questionnaire	Face-to-face interviews	Cross-sectional study	50 individuals were aged > 45 years old (36.2%), 135 individuals (97.8%) had a history of substance abuse in their family, and 66 individuals (40.5%) initiated drug use before age 20. The most common substances were opium and crack with a proportion of 76.8% and 9.4%, respectively. Prevalence of positive HIV and hepatitis B among addicted women were 18.8% (26 persons) and 5.0% (7 persons), respectively. Three (2.1%) of addicted women with HIV also had HBV. The most commonly HIV transmission were drug injections (30.7%) and unprotected sex (11.5%).
17	Haque et al. (2018)	2018	Bangladesh	(1) To investigate the knowledge and awareness of HIV among married women in Bangladesh<br>(2) To assess the extent of HIV knowledge among women in Bangladesh.<br>(3) To identify the responsible factors for changing knowledge, attitudes and intensity about HIV/AIDS, make a comparison between current and previous extent of HIV knowledge, and find the spans where further improvement is required to enhance the consciousness of HIV knowledge.	Cohort: Ever-married women had heard of HIV/AIDS (2014 Bangladesh Demographic and Health Survey (BDHS))<br><br>Size: n = 12593	HIV/AIDS	Knowledge, awareness	Self-administered questionnaire	Face-to-face interviews	Cross-sectional study	62% of the respondents had an adequate knowledge and consciousness about the HIV/AIDS.
18	Huda et al. (2022)	2022	Bangladesh	To investigate the prevalence of STI symptoms among ever-married women in Bangladesh and the associations of STI symptoms with various	Cohort: Ever-married women of repdocutive age (15 - 49 y/o) from Bangladesh in 2007, 2011 and 2014 Bangladesh	STIs	Prevalence, demographic, socioeconomic and behavioural risk factors	Bangladesh Demographic and Health Surveys (BDHS).	Available dataset	Cross-sectional study	The prevalence of abnormal genital discharge and genital sores/ulcers among ever-married women aged 15–49 years was 10% and 6%, respectively. Multivariable analysis revealed that for women aged 25–34 years, those who used contraceptives and married earlier had an increased likelihood of STI symptoms.

				demographic, socioeconomic, and behavioral risk factors using the most recent available data (2007, 2011, and 2014) of the Bangladesh Demographic and Health Surveys (BDHS)	Demographic and Health Survey (BDHS) dataset<br><br>Size: n = 41777						
19	Hue et al. (2020)	2020	Chongqing, China	To explore the prevalence and changing trends of HIV, syphilis, hepatitis C virus (HCV) infections and risk behaviours among female sex workers (FSWs) and to provide reference and theoretical basis for formulating targeted interventions.	Cohort: FSWs were included if they (1) were aged ≥16 years, (2) provided commercial sex for money or goods during the previous month and (3) were willing to participate in the survey and could provide verbal informed consent<br><br>Size: n = 16791	HIV, syphilis, hepatitis C virus (HCV) infections	Prevalence, changing trends, behavioural factors/risk behaviours	Anonymous, standard interviewer-administered and face-to-face question-naire	Face-to-face interviews	Cross-sectional study	HIV infection was correlated with no condom use in the last commercial sex (adjusted OR (aOR) 3.48, 95%CI 1.90 to 6.37) and syphilis infection (aOR 4.88, 95%CI 1.95 to 12.18). Syphilis infection was correlated with inconsistent condom use (aOR 1.30, 95%CI 1.02 to 1.65), HIV infection (aOR 5.88, 95%CI 2.40 to 14.41), HCV infection (aOR 7.68, 95%CI 4.37 to 13.49) and sexually transmitted infection (STI) diagnosis in the past year (aOR 3.81, 95%CI 2.40 to 6.03). HCV infection was associated with injecting drug use (aOR 8.91, 95%CI 4.45 to 17.86) and syphilis infection (aOR 7.88, 95%CI 4.49 to 13.83).
20	Iqbaletal2019	2019	Pakistan	aims to explore the effects of these determinants, related to socio-demographic characteristics and autonomy, on women's overall knowledge and attitudes regarding HIV/AIDS in Pakistan.	Cohort: ever-married women<br><br>Size: 13558	HIV/AIDS transmission	Determinants of attitudes and knowledge	Self-administered	questionnaire	cross-sectional study	the majority have good overall knowledge of HIV/AIDS and more than half have positive attitudes towards people living with AIDS. women residing in urban areas, having at least secondary-level education, with high autonomy, belonging to the richest wealth quintile and having exposure to mass media had high overall knowledge and positive attitudes towards people living with AIDS.
21	Irfanetal2019	2019	Pakistan	aimed to assess the misconceptions and attitude regarding HIV transmission and pre-vention among antenatal mothers, with regards to mother-to-child-transmission (MTCT) in three tertiary care hospitals of Karachi.	Cohort: pregnant women<br><br>Size: 350	HIV transmission	knowledge and attitude	Structured questionnaire	questionnaire	cross-sectional study	Of the 350 pregnant females participating, around 66.7% (N = 232) were urban residents, lying in their mid-twenties and over 86% (N = 303) were Muslims. Over 14.3% (N = 52) of the participants had full knowledge regarding MTCT of HIV, with only 6% of the respondents having full knowledge related to PMTCT of HIV. Significant associations were found between knowledge about MTCT and residence, education level and expected response from the partner. While only 17% (60 out of 350) women agreed to invite their partner for testing, 84% showed an interest in looking after an HIV infected family member and 49% believed that all pregnant females should be tested for HIV.
22	Jahangir et al	2021	Iran	To determine the knowledge, attitudes and behaviours of pregnant women regarding HIV	Cohort: pregnant women<br><br>Size: 200	HIV	Knowledge, attitudes	Questionnaire	Interview	Cross-sectional study	The majority knew that mother-to-child HIv transmission during pregnancy was possible (82.5%)<br>Fewer than half knew that HIV can be

				prevention and rapid HIV tests						transmitted through breastfeeding (48.2%)<br>22.5% knew that a Cesarean section for HIV-positive mothers is recommended<br><br>Overall, the knowledge of mothers regarding HIV transmission is low
23	Jiang et al	2021	China	To characterise low-tier female sex workers who engage in commercial sex with old male clients	Cohort: low-tier female sex workers<br><br>Size: 2647	STDs	Risky behaviour	Questionnaire	Survey	Cross-sectional study	44.0% had engaged in commercial sex with older men clients<br><br>Low-tier female sex workers who:<br>1. work out of roadside shops<br>2. had engaged in sex work for longer<br>3. have a larger number of clients<br>4. had engaged in anal or oral sex during the previous month<br>5. are currrently using contraception measures<br>6. had STI symptoms<br>7. had been exposed to HIV prevention services during the previous 6 months<br>--were more likely to engage in commercial sex with older men clients<br><br>Female sex workers who:<br>1. have a higher level of education<br>2. work out of small venues other than streets, hair salons and roadside shops<br>3. who charged more for commercial sex<br>4. had sex with young clients during the previous months<br>5. had seen a doctor during the previous 6 months<br>--were less likely to engage in commercial sex with older men clients
24	Jommaroeng et al	2019	Thailand	To investigate the effectiveness of the national HIV prevention outreach program for men who have sex with men (MSM) and transgender women (TGW)	Cohort: MSM and TGW<br><br>Size: 16539	HIV	Risky behaviour, awareness	Questionnaire	Interview	Mixed-methods study - qualitative study and cross-sectional study	The program was found to affect changes in:<br>1. condom use with steady partners<br>2. condom use with casual partners<br>3. water-based lubricant use<br>4. HIV testing and counselling uptake<br>5. STI screening uptake<br><br>Gender identity and province of outreach are associated with condom use with steady partners<br>Gender identity and sex work are associated with the use of lubricant
25	Jozani et al. (2019)	2019	Tehran, Iran	To assess knowledge, attitude and practice (KAP) towards HIV/AIDS and to perform HIV, HBV, HCV and HSV2 serosurveys among girls	Cohort: girls from dysfunctional families in Tehran<br><br>Size: 188	HIV/AIDS, HBV, HCV & HSV2	Knowledge, attitude & practice (KAP)	Self-administered, semi-structured standard questionnaire [Family Health	Sero-survey	Cross-sectional study	N = 98 (52.2%): Had good knowledge about HIV/AIDS before educational intervention, then N = 133 (70.7%) after educational intervention<br><br>N = 177 (94.1%): Willing to be tested for HIV

				from dysfunctional families in Tehran, Iran				International (FHI), HIV-AIDS-STD Behavioural Surveillance Survey (BSS) for use among female sex workers (FSWs)/BSS-JAMAICA-1999]			before counseling<br><br>No positive result for HIV, HBV, HCV and HSV2 serosurvey
26	Kakchapati	2018	Nepal	to assess HIV awareness and safe sexual behavior among the female sex workers (FSWs) in the Kathmandu valley of Nepal.	COHORT: FSW<br>SIZE: 2093	HIV	awareness & risky behavior	Integrated Biological and Behavioral Surveillance	interview	cross-sectional	FSWs who had consistent condom use with nonpaying partners, had a HIV test, met peer educators (PEs) and visited the DIC in the last year had a higher chance of condom use with clients.
27	KhalidMartin	2018	Pakistan	whether network operators increase the likelihood of condom use among female and transgender CSWs	COHORT: CSW<br>SIZE: 2326	HIV	Risky behavior	Integrated Behavioral and Biological Survey.	survey	cross-sectional	transgender CSWs recruiting clients through network operators had higher odds of consistently using condoms compared with female CSWs recruiting clients through another source
28	Khan	2021	Bangladesh	to examine the knowledge about HIV transmission among Rohingya refugee women	COHORT: Rohingya refugee women<br>SIZE: 508	HIV	knowledge	Demographic and Health Survey	interview	cross-sectional	knowledge of HIV transmission among Rohingya women was significantly lower than among women in Bangladesh and Myanmar.
29	Khuat	2018	Vietnam	examines the pre- and post-clinical issues in HIV care and treatment for women and girls of high-risk population groups—namely sex workers, injecting drug users, women living with HIV, primary sexual partners of people living with HIV, adolescent girls who are children of these groups, and migrant young girls and women	COHORT: women & girls<br>SIZE: 241	HIV	knowledge	own	survey & interview	cross sectional	18.9% women living with HIV disclosed their infection status, while 37.8% gave no information at the most recent prenatal care visit.<br>level of knowledge and proper practices of sexual and reproductive health (SRH) care remains limited.<br>very low usage of condoms
30	Khumaidi	2021	Indonesia	evaluate the relationship between condom use-negotiation, alcohol consumption and HIV-risk sexual behavior among FSW in Kupang.	COHORT: FSW<br>SIZE: 125	HIV	Risky behavior	safe sexual behavior questionnaire (SSBQ), Condom Influence Strategy Questionnaire (CISQ), Alcohol Use Disorders	self-administered questionnaires.	cross-sectional	majority of FSWs reported had low negotiation condom use with their clients, about 77.6% reported high levels of alcohol consumption and 64.8% had high-risk sexual behavior.<br>Condom-use negotiation and alcohol consumption were found to be significantly associated with HIV-risk sexual behavior.

								Identification Test (AUDIT)			
31	Kurniawati	2021	Indonesia	determine the characteristics, sources of information, and housewives' knowledge about HIV / AIDS and PMTCT.	COHORT: housewives SIZE: 32	HIV	knowledge	previous literature	survey	housewives living in Kulon Progo.	most respondents are of 35 years, 50% are secondary educated, 65% of the respondents have less knowledge about HIV/AIDS and PMTCT. The respondents get information sources on HIV/AIDS, from electronic media, health workers and print media.
32	Larkieral2021	2021	Iran	to understand the experience of violence among HIV negative married women in heterosexual serodiscordant relationships.	Cohort: married women<br><br>Size: 15	HIV	qualitative inquiry	Semi-structured interview	qualitative description	qualitative study	The main overarching theme emerged entitled: life loaded with threat and vulnerability. This theme consisted of four categories of self-directed violence, intimate partner violence, cultural violence and structural violence.
33	Manathunge	2020	Sri Lanka	provide evidence on HIV, syphilis and hepatitis B (HBV) prevalence, sexual risk behaviours and utilisation of HIV prevention interventions among female sex workers (FSW)	COHORT: FSW SIZE: 458	HIV & STI	Risky behavior	own	questionnaire	cross-sectional	90% of FSW used condom at last sex with a client in both Colombo and Galle, but considerably less in Kandy. The commonest reasons for never testing for HIV was not knowing where to test
34	Maqsood	2021	Pakistan	to investigate the determinants related to overall knowledge about and behaviour in relation to HBV and HCV amongst married women	COHORT: married women SIZE: 12,364	HBV, HBC	knowledge & risky behavior	Demographic and Health Survey	questionnaire	cross-sectional	(88.3%) have heard of HBV and HCV. 34.8% had comprehensive knowledge about how to avoid HBV and HCV.
35	Mariani	2021	Indonesia	the relationship between knowledge and personal hygiene and the incidence of sexually transmitted diseases in adolescents	COHORT: married women SIZE: 12,364	STD	knowledge	own	questionnaire	case–control	individual hygiene and the incidence of sexually transmitted diseases (STDs) in adolescents was significant
36	Miankouhi	2018	Iran	awareness of known women with high-risk sexual status about sexually transmitted infections (STIs)	COHORT: FSW SIZE: 173	STI	knowledge	own	questionnaires	cross-sectional	knowledge on STI is high among FSW but knowledge level was low about the symptoms, treatment, and transmission ways of HPV.
37	Mo	2019	HK	prevalence of HIV testing behavior and intention, and identified factors associated with HIV testing intention among women engaging in compensated dating in Hong Kong.	COHORT: women SIZE: 183	HIV	attitude	own	online survey	cross-sectional	that having ever received HIV testing, attitudes towards HIV testing, subjective norm, perceived behavioral control and perceived discrimination from health care workers significantly predicted intention to take up HIV testing.

38	Nematollahi et al. (2022)	2022	Iran	To assess sexual behaviors and vulnerability of transgender women to STIs including HIV	Cohort: Transgender women recruited from August 2019 to March 2020 in Iran at "Support center for Iranian transgender" and "Shiraz Forensic Medicine" where transgender individuals refer to follow the steps of gender afrmation<br><br>Size: n = 127	STIs, HIV	Sexual behaviours, vulnerability	Researcher-made questionnaire	Survey	Cross-sectional study	92.1% of participants were single with experience of sex and 59.3% had one sex partner in the last 2 years. 96.9% of the participants were heterosexual with 67.2% reporting experiencing orgasm in at least 50% of their sexual intercourse. However, 42.5% reported sexual pain and the same percentage reported low or very low sexual satisfaction. About half of the participants used condoms occasionally during sex (48.7%) and the most important reason for not using condoms in most cases was not having a condom (37.9%). Some of participants had little knowledge of the symptoms (33.9%) and complications (44.1%) of STIs. Although 87.4% and 72.4% of participants had never been tested for a STI and HIV, 1.6% were HIV positive and 18.1% had a history of STIs. Also, 26% of people had undergone vaginoplasty and a signifcant association was observed between vaginoplasty with sexual satisfaction (p < 0.01).
39	Noe et al. (2018)	2018	Taunggyi Township, Myanmar	To examine 1) the knowledge and perception of communication on sexual and reproductive health issues, and 2) sexual and reproductive health communication barriers between mothers and adolescent girls.	Cohort: Mother-daughter pairs participated from four Taunggyi Township wards<br><br>Size: n = 112	Sexual and reproductive health (SRH) communication between mother and adolescent girl	Knowledge, perception, barrier	Semi-structured questionnaire	Face-to-face interviews	Community-based cross-sectional study	More than half of both mother and adolescent girls had negative perceptions of communication on SRH issues. Only 2.7% of girls discussed SRH issues with their mothers more than four times in the last six months.
40	Pei Et Al	2020	China	This study investigated the sexual behaviors and the related social determinants of health for HIV infection in Yi women of childbearing age in this area.	Cohort : Yi Women<br>Size : 800	HIV	Sexual behaviours	ELISA	Face to face interview	Cross-sectional study	Path analysis of the risk factors revealed that casual sex (0.152) and number of sex partners (0.152) were directly associated with HIV infection. Furthermore,<br>education level (0.057), out-migrating for work (0.032), sense of self-worth (0.024) and number of sex partners (0.079) were indirectly related to HIV infection and mediated by casual sex and multiple sexual partners.
41	Pradnyani Et Al	2019	Indonesia	The purpose of this study was to characterize Indonesian women's knowledge of HIV/AIDS and to investigate the effects of socio-demographic characteristics thereupon	Cohort : Women in Indonesia<br>Size : 34984	HIV , AIDS	Knowledge	Secondary data anaylysis from the IDHS in 2012	Face to face interview	Cross-sectional study	All socio-demographic characteristics except marital status were related to knowledge of HIV/AIDS among Indonesian women in the univariate analysis (p<0.05). Multivariate analysis revealed that only age group, education level, location of residence, and wealth index were related to Indonesian women's knowledge of HIV/AIDS

				with the goal of supporting the prevention and early detection of HIV/AIDS.							
42	Ranjan	2019	India	describe the interpersonal, social, and cultural context of the reported behaviors and information related to marriage and sexual practices commonly prevalent in the rural areas.	COHORT: wives of migrant workers<br>SIZE: 24	HIV	risky behavior	own	interview	cross-sectional	premarital sex was more common in boys than girls.<br>males in villages had many sex partners, risky behaviors as perceived by the participants were living away from the spouse, having a relationship with other women outside the village, visiting a CSW.
43	Rutledge	2018	Malaysia	correlates of recent HIV testing among TW	COHORT: TW<br>SIZE: 199	HIV	knowledge	own	survey	cross-sectional	41.7% of TW reported having ever been tested for HIV. Having a primary care provider (PCP), being 26–40 years of age, and having higher mental health functioning were positively associated with recent HIV testing.
44	Saeieh et al. (2018)	2018	Iran	Study on non-disclosure experience in Iranian Women regarding HIV status.	Cohort: HIV-infected women with riksy behavior<br><br>Size: 15	HIV	Disclosure	Interview questions	Semi-structured interview	Qualitative study	Women with HIV have a lot of problems in disclosing their disease status.<br><br>Disclosure of HIV status was classified based on the experiences of the participants into 2 main categories: fear of rejection and community construction of HIV.
45	Samal et al. (2019)	2019	India	Find out the prevalence of HBV infection and associated risk factors among healthy pregnant ladies in Southern Odisha.	Cohort: Healthy pregnant ladies aged 18-45 years old<br><br>Size: 3230	Hepatitis B	Awareness, risky behavior	ELISA	Hepatitis B surface antigen (HBsAg) test	Prospective, observational study	4.64% were tested positive for HBsAg.
46	Seekaew et al. (2019)	2019	Thailand	Investigate the discordance between self-perceived HIV risk and actual risk among MSM and transgender women in Thailand.	Cohort: MSM, transgender women<br><br>Size: 2613	HIV	Perception	Self administered questionaire	Survey, HIV / STI test	Cross-sectional study	Over 80% of the participants perceived themselves as having low HIV risk even though they actually undertook considerable HIV-related risks.
47	Shan et al. (2018)	2018	China	Understand the risk behaviors and factors associated with HIV infection among transgender women in two cities in China.	Cohort: Transgender women from Shanghai and Tianjin.<br><br>Size: 498	HIV	Risky behavior	Structured questionaire	Survey	Cross-sectional study	Transgender women bear a high HIV burden in the two Chinese cities.<br><br>Those involved in commercial sex tended to have inconsistent condom use, leading to high risk of HIV infection.<br><br>Substance use was an independent risk factor of HIV infection by increasing sexual activities and unprotected sex, which indicated an aggravated and complex situation with possible interacting syndemic factors that could cumulatively facilitate sexual risk behaviours and HIV infection in transgender women.

48	Shan et al. (2022)	2022	China	Estimate HIV incidence and distinguish risk factors of HIV acquisition among transgender women and cisgender MSM.	Cohort: Transgender women and cisgender MSM from Shanghai and Tianjin.<br><br>Size: 1056	HIV	Risky behavior	Structured questionaire	Survey, HIV rapid test	Open cohort study	Compared to cis-MSM, transgender women were at higher risk of HIV acquisition.
49	Shrestha et al. (2020)	2020	Malaysia	Assess willingness to use HIV self-testing (HIVST) among transgender women in Malaysia.	Cohort: Transgender women<br><br>Size: 361	HIV	Perception	Questionaire	Survey	Cross-sectional study	Transgender women were moderately willing to use HIVST.
50	Sinha et al. (2020)	2020	India	Determine the sociodemographic profile of the female sex workers (FSW) under the study, assess their knowledge and attitude towards HIV/AIDS, and find out their sexual practices.	Cohort: FSW<br><br>Size: 90	HIV	Knowledge, attitude	Interview questions	Structured interview, focused group discussion	Cross-sectional study	Regarding knowledge of the study population 90% had heard the name of HIV/AIDS.<br><br>While assessing the attitude about HIV/AIDS in the study population, 82.2% of them were worried about infected with HIV, 32.2% of the study population surprisingly said that people with HIV/AIDS were of improper characters.<br><br>Regarding sexual activity, 76.7% had sexual activity more than 3 times/week and 97.8% used condom persistently in last one year.
51	Son et al. (2020)	2020	Vietnam	Assess the trends and associated factors of comprehensive knowledge about HIV among women in Vietnam.	Cohort: Women aged 15-49<br><br>Size: 9252	HIV	Knowledge	Vietnam Multiple Indicator Cluster Surveys (MICSs) 2000, 2006, 2011, 2014	Survey	Secondary analysis	Comprehensive knowledge about HIV among women in Vietnam increased from 2000 to 2014, but it was still relatively low.
52	Stoicescu et al. (2018)	2018	Indonesia	Examine associations and additive efects of different forms of intimate partner violence (IPV) victimization (psychological, physical and/or injurious, and sexual) on HIV sexual risk behavior among women who inject drugs in Indonesia.	Cohort: Women who inject drugs from urban areas<br><br>Size: 731	HIV	Risky behavior	Interview questions	Interview	Cross-sectional study	All three forms of IPV were associated with elevated odds of engaging in sexual risk behaviors.<br><br>There were statistically signifcant positive associations between engaging in sexual risk behavior and the following covariates in all three multivariate models: crystal meth use, HIV-positive status, younger age, lower than high school educational attainment, and not being currently married.
53	Storm et al. (2020)	2020	Nepal	Assess the prevalence of HIV and syphilis, and how individual and socio-structural factors influence sexual risk behaviour and health care service uptake, among MSM and transgender women in the Terai highway districts of Nepal.	Cohort: MSM, transgender women<br><br>Size: 340	HIV, syphilis	Risky behavior	Integrated Biological and Behavioural Surveillance (IBBS) survey	Survey	Secondary analysis	The prevalence of HIV among MSM was 5%, whereas it was 13% among transgender women.<br>The prevalence of active syphilis was 4% in the MSM group and 11% among transgender women.<br><br>Respondents that identified as MSM and had an income above 10,000 Nepalese rupees had a lower likelihood of using a condom in the last sexual encounter compared to

											respondents that earned less.<br><br>Controlling for the other covariates in the model, being forced by family to marry a female was associated with lack of condom use during the last sexual encounter among MSM.<br><br>Among both MSM and transgender women,having visited an outreach centre was positively associated with condom use during the most recent sexual encounter.<br><br>Multivariable regression revealed that being away from home the past month was associated with increased condom use among transgender women.<br><br>Having a moderate response to unfair treatment was associated with a decreased odds of condom use compared to a passive response.
54	Tuo et al. (2020)	2020	Cambod ia	To explore the HIV prevalence and identify risk factors associated with HIV infection among female entertainment workers (FEWs) in Cambodia	Cohort: Women who were (1) working in the entertainment establishments or as freelance sex workers; (2) aged at least 18 years; (3) sexually active, defined as having had vaginal or anal sexual intercourse with at least one man in the past 12 months; (4) able to communicate in Khmer; (5) able and willing to provide written informed consent; and (6) willing to be physically present at the study site for an interview and HIV testing<br><br>Size: n = 3149	HIV infection	Prevalence, risk factors	Structured questionnaire	Face-to-face interviews	Two-stage cluster sampling method national biological and behavioural survey-based study	The odds of HIV infection were significantly higher among FEWs in the age group of 31 to 35 (AOR 2.72, 95% CI 1.36–8.25) and 36 or older (AOR 3.62, 95% CI 1.89–10.55); FEWs who were not married but living with a sexual partner (AOR 3.00, 95% CI 1.16–7.79); FEWs who had at least ten years of formal education (AOR 0.32, 95% CI 0.17–0.83); FEWs who reported having abnormal vaginal discharge (AOR 3.51, 95% CI 1.12–9.01), genital ulcers or sores (AOR 2.06, 95% CI 1.09–3.17), and genital warts (AOR 2.89, 95% CI 1.44–6.33) in the past three months; and FEWs who reported using illicit drugs (AOR 3.28, 95% CI 1.20–4.27) than their respective reference group. The odds of HIV infection were significantly lower among FEWs working in karaoke bars (AOR 0.26, 95% CI 0.14–0.50) and beer gardens (AOR 0.17, 95% CI 0.09–0.54) than among freelance FEWs.
55	Upadhyay et al	2020	India	To evalute the knowledge, attitude and perception of HIV/AIDS among antenatal women	Cohort: pregnant women<br><br>Size: 400	HIV	Knowledge, attitude	Questionnaire	Interview	Cross-sectional study	26% of women were totally unaware of any entity like HIV<br>44% did not know the most common way of spread of HIV

				and to correlate them with their socio-demographic profile						Half knew the correct preventive mesasures for HIV/AIDS<br>54% knew about mother to child transmission, but only 24% knew about its transmission through breast milk<br><br>Knowledge and attitude was found to be significantly improving with socioeconomic status
56	Virdausi et al	2022	Indonesia	To analyze socio-economic and demographic factors related to knowledge and attitude of HIV/AIDS	Cohort: women aged 15-49 years old<br><br>Size: 25895	HIV	Knowledge, attitudes	Questionnaire based on DHS standards<br><br>https://dhsprogram.com/pubs/pdf/DHSQ8/DHS8 _Womans_QRE _EN_8Apr2022 _DHSQ8.pdf	Survey	Cross-sectional study	Women's age, education level, wealth quintile, residential area and region, access to information, owning cell phones and autonomy were significantly associated with positive knowledge and attitudes toward HIV/AIDS<br><br>88.74% had a high level of knowledge, 60.28% had negative attitudes towards PLWHA
57	Wangetal2021	2021	China	described IPV, sexual risk behaviors, HIV, and sexually transmitted infection (STIs) testing rates and results, and investigated the pathways that link IPV to HIV infection among this population.	Cohort: transwomen<br><br>Size: 199	HIV testing	associations with sexual risk behaviours and HIV testing	ELISA	collect blood samples	cross-sectional study	More than half of the respondents reported IPV (57.3%), and the prevalence of unprotected sex ranged from 51.9% (with sex workers) to 87.8% (oral sex); 85.9% had ever tested for HIVand 49.3% for other STIs. Self-reported positivity results were as follows: HIV (2.3%), herpes simplex virus type 2 (8.3%), gonorrhea (18.8%), and syphilis (17.8%). Laboratory-confirmed positivity values were 5.0% for HIV and 6.5% for syphilis. Respondents with a history of IPV were significantly less likely to report HIV testing in the past 12 months
58	Wansometal2021	2021	Thailand	We measured Human Immunodeficiency (HIV) incidence, retention, and assessed risk factors for seroconversion among two previously unreported cohorts of men who have sex with men (MSM) and Transgender Women (TGW) in Bangkok, Thailand between 2017 and 2019.	Cohort: MSM and transgender women<br><br>Size: 1184	HIV sero-conversion	risk factors	HIV and syphilis testing and computer-based behavioral	questionnaire	prospective cohort design	Over the 18-month study, visit retention was 93·4% and HIV incidence was 3·73 per 100 person-years. Utilizing survival regression tree modeling, those who were 1820 years of age, reported sexual attraction to mostly or only men, and had five or more lifetime sexual partners were 4·9 times more likely to seroconvert compared to other cohort participants. Factors associated with HIV incidence utilizing Cox pH regression included sexual attraction to mostly or only men, younger age, five or greater lifetime sexual partners, inconsistent condom use with casual partners, and prior HIV testing
59	Wilsonetal2021	2021	China, Myammar, Thailand	to examine the experiences of FSWs in the Yunnan to better understand the risk of infection and the	Cohort: Female sex workers<br><br>Size: 20	HIV risks	eperiences	semi-structured	interviews	Thematic analysis	The fndings showed that FSWs' primary source for HIV information was gynecologists, with few visiting the local HIV charity Red Ribbon. FSWs reported infrequent visits for check-ups with some seeing a gynecologist

				potential for transmission of HIV.							once a year. FSWs felt that the onus was on them to prevent STI/HIV infection by using a condom during sex, regardless of their ability to negotiate use. FSWs were also reluctant to see a gynecologists for treatment. Instead, they resorted to douching as a way of preventing HIV/STIs and treating vaginal health problems, such as leucorrhea. Most FSWs worked without the infuence of alcohol and drugs. A small number of FSWs reported heroin addiction and injecting drug use.
60	Wuetal2019	2019	China	examine current sex behaviors, relationships, types of partners, and unprotected sex among different sexual partners of HIV positive CFSWs, in order to understand the reasons for unsafe sex behavior.	Cohort: female sex workers<br><br>Size: 327	HIV	risk behaviours	face to face	questionnaire	mixed methods	Among the participants of the quantitative survey, 41.6% (136/327) had sex with regular sexual partners only in the past 6 months, of whom 64.0% (87/136) had consistent condom use; 27.5% (90/327) of the participants had sex with irregular sexual partners in the past 6 months, of which, 46.7% (42/90) had consistent condom use. The qualitative study suggested that HIV positive sero-status, willingness to protect their spouses or regular sexual partners, and lacking a sense of responsibility to protect their commercial and casual sexual partners, influence CFSWs' sexual behaviors.
61	Yan Et Al	2021	China	This study is aimed to assess tran's women awareness of and willingness to use PrEP.	Cohort : trans women in China<br>Size : 222	HIV	Awareness and willingness to use HIV PrEP	Self-administered web-based questionnaire	Survey	Cross-sectional study	Of 222 HIV-negative/unknown serostatus trans women, 33.3% were aware of PrEP and 49.1% were willing to use PrEP. PrEP awareness was associated with a university degree or above and not using alcohol with sex. Willingness to use PrEP was higher among trans women with one or multiple sexual partners<br>compared to those with no partners.
62	Yi Et Al	2018	Cambodia	This study aimed to identify factors associated with recent HIV testing among FEWs in Cambodia to inform future prevention activities.	Cohort : Female Entertainment workers<br>Size : 667	HIV , AIDS	Sexual behaviours , knowledges , HIV testing attitudes	Structured questionnaire	Face to face interviews	Cross-sectional study	Of total, 81.7% reported ever having had an HIV test, and 52.8% had at least one test in the past six months.<br><br>After adjustment for other covariates, factors independently associated with recent HIV test-<br>ing included living in Phnom Penh , having received HIV education in the past six months , disagreeing with a statement that 'I would rather not know if I have HIV' , agreeing with a statement that 'getting tested for HIV helps people feel better' and not using a condom in the last sexual intercourse with a non-commercial partner
63	Yu Et Al	2020	Vietnam	This consecutive cross-sectional study examines demographic and	Cohort : Female Sex Workers<br>Size : 303	HIV , STI	Risk behaviours	Data from China's National HIV	Survey	Cross-sectional study	Vietnamese FSWs were younger, had attained lower education levels, were highly mobile, more likely to report using drugs, and were

				behavioral factors associated with HIV/STI infection, Hepatitis C (HCV) and other sexually transmitted infections (STIs) among Vietnamese female sex workers (FSW), a vulnerable population who cross into Guangxi, China.	Vietnamese and 4348 Chinese FSW			Sentinel Surveilance			more vulnerable to HIV/STIs. Younger age, less educational attainment, shorter time in their current working location, no voluntary HIV testing in the last year, greater drug use, and not using condoms for all commercial sex in the last month were associated with higher HIV/STIs.
64	Zakaria et al	2020	Bangladesh	To explore the level of knowledge, attitudes and practice of sexual and reproductive health among college-going older adolescent girls	Cohort: older adolescent girls (age 16-18)<br><br>Size: 792	STDs	Knowledge	Self-administered questionnaire	Survey	Cross-sectional study	54.8% had complete information on the item 'the carrier of the STIs may unintentionally transmit the virus to its partner'<br><br>40% responded correctly regarding whether HIV can be spready through mosquitoes and fleas<br>25.4% knew that HIV is not spread by an infected person coughing and sneezing
65	Zarei et al	2018	Iran	To investigate the knowledge of Iranian women about HIV/AIDs and whether they had accepting attitudes towards people living with HIV, and identify factors correlated with their knowledge and attitudes	Cohort: Iranian women at least 15 years of age<br><br>Size: 44921	HIV	Knowledge, attitudes	Questionnaire based on the Multiple Indicators Cluster Survey (national survey conducted in 2015)	Survey	Cross-sectional study (unsure)	The majority (79.0%) had heard about HIV/AIDS, but only 19.1% had a comprehensive knowledge<br>15.4% had accepting attitudes toward people with HIV<br><br>Being older, married, more highly educated and wealthier were factors associated with having more comprehensive knowledge of HIV/AIDS<br>Living in urban areas was associated with having more positive attitudes towards people with HIV
66	Zhou et al	2020	China	To investigate the prevalence of oral sex and its associated factors among low-tier female sex workers (FSWs)	Cohort: low-tier female sex workers<br><br>Size: 2645	STDs	Risky behaviour	Structured questionnaire	Survey	Cross-sectional study	Oral sex is related to:<br>1. being unmarried<br>2. low income<br>3. early initiation of commercial sex<br>4. having conducted commercial sex in more counties<br>5. longer duration of commercial sex<br>6. larger number of clients<br>7. ever having engaged in anal sex<br>8. less use of condoms and oral contraceptives during the previous month<br>9. low rate of adoption for contraception at the present time<br>10. STI-related symptoms during the previous half-year
67	Zin et al	2019	Malaysia	To evaluate the knowledge, attitude and practice of STDs among selected inmates of women shelter homes	Cohort: inmates of women shelter homes<br><br>Size: 60	STDs	Knowledge, attitude	Self-administered questionnaire	Survey	Cross-sectional study	The majority have heard of HIV/AIDS (95%) but with respect to other STDs was less well known<br><br>Knowledge level of participants on STDs were

											classified into 3 groups:<br>1. high knowledge (33.3%)<br>2. medium knowledge (35.05)<br>3. low knowledge (31.7%)<br><br>Mean score for attitude was 23.1 out of 25<br>43% have three or more sexual partners within 3 years<br><br>Knowledge level was not influenced by age and socioeconomic status, but attitude was influenced by age
