# Supplementary material for: Understanding Women’s Knowledge, Awareness, and Perceptions of STIs/STDs in Asia: A Scoping Review
Source: Healthcare (Basel). 2023 Sep 28;11(19):2643. doi: 10.3390/healthcare11192643 (PMC10572356; doi:10.3390/healthcare11192643)
Supplement: Supplementary file 1 [file healthcare-11-02643-s001.zip › Supplementary Table 2.pdf]

| No | Author | Year | Factors | Sociodemographic (if any) | Education | Age | Geographical | Marital Status | Socioeconomic | Religion | Occupation | Knowledge | Awareness | Perception |
|---|---|---|---|---|---|---|---|---|---|---|---|---|---|---|
| 1 | Abadi et al. | 2018 | Occupation , the first time FSW had a first sexual partner may be - a/w with high risk behaviours , however educational level may play a role but have not much significance. | Occupation , Educational level , Marital status | | < 30 | | | L | | FSW | | | |
| 2 | Allahqoli Et Al | 2018 | N/A | N/A | | | | | | Y | | | 13.96 ± 8.7 | |
| 3 | Arends Et Al | 2019 | NA | Educational status , occupational status , marital status , risky behaviours , criminal history | | > 30 | | | L | | | | | |
| 4 | Ayuttacorn Et Al | 2019 | Stgimatised fear, gender norms | Age , Place of birth , Educational status , Religion , Occupational Status , Sexual Activities | L | > 30 | | M | L | Y | Emp | | | |
| 5 | Biswas et al. (2020) | 2020 | FSWs who are widowed/divorced/separated, with sex work the only income source and used injecting drugs for nonmedial purpose had a higher risk of catching HIV. | Demographic, sex work characteristics, marital status, use of drug | L | < 30 | U | D | L | | Sex workers | | | |
| 6 | Cempaka et al. | 2020 | | | L | > 30 | | M | | | Emp | | 16.40% | |
| 7 | Chakrapani et al | 2021 | occupation | | L | > 30 | U | | L | | Sex workers | | 17.10% | |
| 8 | Damasetal2021 | 2021 | women who enrolled in national programmes such as family planning | | L | < 30 | R | | L | | | | | |
| 9 | Devarayasamudrametal2018 | 2018 | structured teaching programme | | L | < 30 | | M | L | Y | Emp | 67.30 % | | 77% |
| 10 | Efendi et al. (2020) | 2020 | Women aged 30-34 years old had 2.2 times higher knowledge about HIV compared to older women.<br><br>Married women, living in rural area, with a lower level of education, reported to have limited to no access to HIV-related information. Thus, had lower knowledge level of HIV. | Age, marital status, place of residence, education level, socioeconomic status, access to information | L | both | U | M | L | | | 53.60 % | | |
| 11 | Emmanual et al. (2020) | 2021 | Being uneducated / illiterate, directly dealing with clients without a pimp, non-use of condom with clients, having sexual contact with people who inject drugs (PWID), and non participation in HIV programmes were factors independently associated with HIV seropositivity. | Education level | L | < 30 | | M | L | | sex workers | | | |

| | | | | | | | | | | | | | | |
|---|---|---|---|---|---|---|---|---|---|---|---|---|---|---|
| 12 | Galka | 2020 | Attitudes & preferences | Chinese ethnicity was significantly negatively associated with willingness to use PrEP. Older TW were less willing to use PrEP | L | > 30 | U | U | L | Y | Sex workers | | | |
| 13 | Gharehghani | 2020 | | | | < 30 | | | | | sex workers | | | |
| 14 | Guida | 2019 | | FSWs were more likely to use condoms if they had better attitudes toward condom use, stronger perceived behavioral control, and higher levels of subjective norms. | | | | | | | Sex workers | | | |
| 15 | Hamdanieh et al. (2021) | 2021 | Comparison between Lebanon and other countries<br>(1) Shortage of educational campaigns in schools and universities due to its sensitivity in the middle eastern culture and its consideration as a taboo in these societies<br>(2) Sexual education not included in curriculum<br>(3) Lack of sexual health services | Socioeconomic status, culture and belief, awareness, educational level | H | < 30 | | U | | | | 8.80% | | |
| 16 | Hamzeh et al. (2019) | 2019 | Middle age, married, good socioeconomic status, and average level of education women have the highest frequency of addiction. | Age, marital status, socioeconomic status, level of education | L | > 30 | | M | L | | | | | |
| 17 | Haque et al. (2018) | 2018 | Respondents with higher education were more aware than those with no education (odds ratio (OR) = 3.56, 95% confidence interval (CI): 2.99–4.23). Moreover, respondents who had access to the mass media were more likely to be aware compared to those who did not have the access (OR = 1.14, 95% CI: 1.04–1.26). | Education status, mass-media access, place of living, working status | L | < 30 | R | M | L | Y | Emp | 62% | | |
| 18 | Huda et al. (2022) | 2022 | Women from the wealthiest wealth quintile and couple's joint decision-making were less likely to have STI symptoms. | Socioeconomic status, couple/spouse co-joint decision | L | < 30 | R | M | L | | U | | | |
| 19 | Hue et al. (2020) | 2020 | Syphilis has an upward trend among FSWs in lower-tier and middle-tier venues (average price of each sex transaction). | Age, marital status, ethnicity, educational level, typology, working time, condom use, injecting drug use and STI diagnosis | L | both | | M | L | | Emp | | | |
| 20 | Iqbaletal2019 | 2019 | residing in urban areas, having at least secondary-level education, with high autonomy, belonging to | | L | | | M | L | | U | 28% | | 55% |

| | | | | | | | | | | | | | | |
|---|---|---|---|---|---|---|---|---|---|---|---|---|---|---|
| | | | the richest wealth quintile and having exposure to mass media | | | | | | | | | | | |
| 21 | Irfanetal2019 | 2019 | education level | | L | < 30 | U | M | L | Y | U | 14.30 % | | 84% |
| 22 | Jahangir et al | 2021 | | | | | | M | L | | Sex workers | 82.50 % | | |
| 23 | Jiang et al | 2021 | Sociodemographic characteristics, behaviour and psychological correlates of female sex workers | | L | < 30 | | M | L | | Sex workers | | | |
| 24 | Jommaroeng et al | 2019 | | | | < 30 | U | | | | | | | |
| 25 | Jozani et al. (2019) | 2019 | Educational intervention (Educational program consisting of 6-hour interactive group discussion on nature of HIV and AIDS, modes of HIV transmission, preventive measures including condom negotiation and promotion) | Educational level | L | | | | | | | 70.70 % | | |
| 26 | Kakchapati | 2018 | | FSWs who had secondary education and visited the drop-in center in the last year had more knowledge and misconceptions about HIV<br>Married FSWs and FSWs who met PEs in the last year were more likely to have condom use with nonpaying partners | L | < 30 | | M | L | | sex workers | | | |
| 27 | KhalidMartin | 2018 | | | L | < 30 | | | L | | sex workers | | | |
| 28 | Khan | 2021 | | knowledge on HIV transmission increases with age at which they were first married.<br>Misconceptions about the modes of HIV transmission high among cohort<br>Women who were involved in work outside their households or those whose husbands were employed were more likely than others to demonstrate relatively good knowledge of transmission.<br>Women with prior formal education were more aware | L | < 30 | | M | | | | | | |
| 29 | Khuat | 2018 | | | L | < 30 | | U | | | | | | |
| 30 | Khumaidi | 2021 | | | L | < 30 | | U | H | | sex workers | | | |

| | | | | | | | | | | | | | | |
|---|---|---|---|---|---|---|---|---|---|---|---|---|---|---|
| 31 | Kurniawati | 2021 | | | L | | | M | | | U | 16% | | |
| 32 | Larkieral2021 | 2021 | - | | | > 30 | | M | L | | U | | | |
| 33 | Manathunge | 2020 | | | | > 30 | | | | | sex workers | | | |
| 34 | Maqsood | 2021 | | women living in urban areas, being older, and having more than 10 years of schooling, reported better knowledge and health behaviours regarding HBV and HCV. | L | < 30 | R | M | L | | U | 34.80 % | | |
| 35 | Mariani | 2021 | | | | | | | | | | 75% | | |
| 36 | Miankouhi | 2018 | | higher education of oneself, the husband and father's occupation were indication of higher STI knowledge | L | < 30 | | D, S, W | L | | U | | 62.1 | |
| 37 | Mo | 2019 | | | H | < 30 | | U | | | | | | |
| 38 | Nematollahi et al. (2022) | 2022 | A signifcant portion of transgender women were not familiar with any STIs signs and symptoms and unfortunately 88.2% did not even consider themselves at risk for HIV infection due to cultural factors and lacking of awareness.<br><br>The high prevalence of STIs and HIV in transgender women compared to the general population indicates their high vulnerability to high-risk sexual behaviors such as unprotected sex and sometimes having multiple sex partners. Similarly, in the studies conducted on transgender women, the prevalence of STIs was between 13 and 21%.<br><br>In this study, about one-third of participants never used condoms during sex, and half of them used it occasionally. In addition, they mentioned that the most common reason for not using a condom was not having one, and given that the majority of participants in the study were in poor financial conditions. | Cultural factors, awareness level, sexual behaviours, socioeconomic status | L | < 30 | | U | L | | U | | | |
| 39 | Noe et al. (2018) | 2018 | The factors found to create SRH communication barriers were higher family incomes (adjusted odd ration [AOR] 2.5, 95% | Socioeconomic status (family income), knowledge/educational | L | < 30 | | U | | Y | | | | |

| | | | | | | | | | | | | | | |
|---|---|---|---|---|---|---|---|---|---|---|---|---|---|---|
| | | | confidence interval [CI] 1.0, 6.2), good knowledge of puberty (AOR 4.5, 95% CI 1.6, 12.5), good knowledge of sexual and reproductive health issues (AOR 4.5, 95% CI 1.8, 11.5), and positive perception of communication (AOR 6.7, 95% CI 2.5, 17.9) among mothers, and good knowledge of contraception (AOR 5.7, 95% CI 1.5, 21.4) and good knowledge of sexually transmitted infections (AOR 2.5, 95% CI 1.0, 6.4) among adolescent girls. | level, perception of communication | | | | | | | | | | |
| 40 | Pei Et Al | 2020 | education, self-worth , outmigrating for work | Age group , marital status , annual household income , educational level , iliteracy, sense of self worth | L | < 30 | | M | L | | | | | |
| 41 | Pradnyani Et Al | 2019 | Occupational status , marital status , educational status , place of residence , household income | Age group , occupational status , marital status , place of residence , wealth index | L | > 30 | U | M | H | | Emp | | | |
| 42 | Ranjan | 2019 | | | | | | | | | | | | |
| 43 | Rutledge | 2018 | | | H | > 30 | U | U | | Y | Emp | | | |
| 44 | Saeieh et al. (2018) | 2018 | Fear of rejection has 2 sub-categories: rejection by partner, and rejection by family.<br><br>Community construction of HIV has 3 sub-categories: discrimination in society, social stigma, and traditional environment for health services. | NA | H | < 30 | | M | | | U | | | |
| 45 | Samal et al. (2019) | 2019 | Ladies wiith multiple sexual partners (57.14%) followed by intravenous drug users (7.88%) were mostly positive for HBsAg.<br><br>The most common age group showing HBsAg positivity was 18-25 years (5.47%).<br><br>Most of the affected women were farmers (8,54%) followed by daily wage labourers (6.5%).<br><br>Maximum frequency of HBsAg positivity was noted among ladies who completed primary education (14.12%). | Age, occupation, education level | L | < 30 | | | | | Emp | | | |
| 46 | Seekaew et al. (2019) | 2019 | Of the 882 MSM and 406 TGW participants who perceived | Sexual orientation, age, place of residence, living | L | < 30 | | U | L | Y | Emp | | | |

| | | | | | | | | | | | | | | |
|---|---|---|---|---|---|---|---|---|---|---|---|---|---|---|
| | | | themselves as having low HIV risk, over 80% reported at least one of the following: tested HIV positive, engaged in condomless sex, tested positive for a sexually transmitted infection, or used amphetamine-type stimulants.<br><br>Living with a male partner, having never tested for HIV, and living in Bangkok and Chiang Mai were associated with increased risk discordance among MSM.<br><br>Living with a male partner, being less than 17 years old at sexual debut, and having a low knowledge score about HIV transmission were associated with increased risk discordance among TGW.<br><br>For TGW, being a sex worker decreased the chance of risk discordance. | with male partner, occupation | | | | | | | | | | |
| 47 | Shan et al. (2018) | 2018 | Nearly half (45.6%) reported having regular partners, and 70.5% had casual partners.<br><br>Regarding condom use, 81.5% reported not always using condoms with stable partners, and 70.9% reported not using condoms with casual partners.<br><br>5.0% had a history of buying sex and 10.2% had a history of selling sex in the past three months.<br><br>40.2% participants had used at least one kind of controlled substance in the past six months. The most commonly used substances were amyl nitrates (rush popper) (99.5%) and 5-MeO-DiPT (20.0%).<br><br>Among rush popper users, 85.4% reported always having sex while on the drug, and 88.9% reported increased sexual pleasure after using the drug. | Place of residence | L | < 30 | | U | L | | Emp | | | |

| | | | | | | | | | | | | | | |
|---|---|---|---|---|---|---|---|---|---|---|---|---|---|---|
| | | | The HIV infection risk factors identified in the study were being located in Shanghai, selling sex in the past three months, and substance use in the past six months. | | | | | | | | | | | |
| 48 | Shan et al. (2022) | 2022 | HIV incidence among transgender women was 4.42 per 100 person-years (PYs), which was signifcantly higher than that of 1.35 per 100 PYs among cis-MSM, demonstrating a threefold higher odds of HIV infection than cis-MSM.<br><br>For transgender women, those lived locally ≤2 years and unprotected anal sex last time were more likely to acquire HIV.<br><br>For cis-MSM, factors associated with HIV acquisition were frequency of anal sex ≥3 times in past one month and unprotected anal sex last time. | Sexual orientation | L | < 30 | | U | | | Emp | | | |
| 49 | Shrestha et al. (2020) | 2020 | Willingness to use HIVST was positively associated with having experienced sexual assault in childhood, having ever used mobile phone or apps to find sex work clients, and having engaged in condomless sex in the past 6 months.<br><br>Living in Kuala Lumpur, having higher number of sex work clients per day, and current depressive symptoms were negatively associated with willingness to use HIVST. | Place of residence | L | > 30 | | U | L | Y | | | | |
| 50 | Sinha et al. (2020) | 2020 | Regarding the mode of the spreading of HIV/AIDS, 76.67% knew that it infects through the vaginal route of sexual activity, nearly 94.4% knew about sharing of the needle and 67.78% knew about the mother-to-child transmission (MTCT).<br>HIV can be transmitted through blood was known to 54.44% of the study population. | NA | L | < 30 | | U | | | Sex workers | 81% | | 92% |

| | | | | | | | | | | | | | | |
|---|---|---|---|---|---|---|---|---|---|---|---|---|---|---|
| | | | 6.67% of the study population had the idea that HIV can be spread by touch while 84.44% of the study population knew that HIV/AIDS can be tested.<br><br>Around 92% of the participants in this study believed that patients with HIV should not be isolated while 93.3% of the participants believed that people with HIV/AIDS should not be deprived of their property but contrarily 6.67% thought that patients with HIV/AIDS should be deprived of their property. | | | | | | | | | | | |
| 51 | Son et al. (2020) | 2020 | Comprehensive knowledge about HIV increased from 26.1% in 2000 to 44.1% in 2011, but it decreased slightly between 2011 and 2014, from 44.1% to 42.4%.<br><br>Increased comprehensive knowledge about HIV was associated with women who had higher education and those in the fourth and fifth quintiles of household wealth in all four rounds of the MICS.<br><br>Comprehensive knowledge about HIV among women was also associated with those who had ever been tested for HIV and those with knowledge of where to be tested for HIV.<br><br>Women in the urban areas were more likely to have higher levels of comprehensive knowledge about HIV as compared to the women in the rural areas in 2000, 2006, and 2011 but not in 2014. | Education level, place of residence, wealth status | H | < 30 | U | | H | Y | | 42.40 % | | |
| 52 | Stoicescu et al. (2018) | 2018 | Women who experienced psychological IPV were nearly three times more likely to engage in sexual risk behavior relative to women who did not experience such abuse.<br><br>Women exposed to physical and/or injurious IPV were at least | HIV status, age, education level, marital status | H | < 30 | | U | H | | Emp | | | |

| | | | | | | | | | | | | | | |
|---|---|---|---|---|---|---|---|---|---|---|---|---|---|---|
| | | | twice more likely to engage in sexual risk behavior.<br><br>Experiencing sexual IPV nearly tripled the odds of engaging in sexual risk behavior.<br><br>Sexual risk behavior was reported by 64.1% of women who did not experience any IPV, but increased to 89.9% among women exposed to all three types.<br><br>Crystal meth use, HIV-positive status, and lower than highschool educational attainment were significantly positively associated with sexual risk behavior.<br>Age and marital status were negatively associated with engaging in sexual risk behavior. | | | | | | | | | | | |
| 53 | Storm et al. (2020) | 2020 | The prevalence of HIV was 2% in the eastern and far-western area, whereas it was slightly higher in the western district (4%).<br>The prevalence of syphilis varied across the regions with a prevalence of 4% in the eastern region, 1% in the western region and 2% in the far-western region.<br><br>When comparing condom use across the different regions of the Terai, 91% of MSM and transgender women in the western region reported to have used a condom in the last sexual intercourse, whereas it was 86% in the far-western region, and 77% in the eastern region.<br><br>When asked who their last sexual partner was 48% MSM and 54% transgender women reported that their last sexual intercourse was with a non-paying male partner.<br><br>A relatively high proportion of MSM (81%) and transgender women (76%) had a high level of knowledge of HIV risk and transmission. | Sexual orientation, place of residence, income | | < 30 | R | U | | | Emp | | | |

| | | | | | | | | | | | | | | |
|---|---|---|---|---|---|---|---|---|---|---|---|---|---|---|
| | | | In the MSM group, 25% reported that their family forced them to marry a female and 30% had been away from their home the past month.<br>Among transgender women, 37% reported that their family forced them to marry a female, and 51% had experienced discrimination in one or more situations.<br><br>The uptake of HIV testing and counselling (HTC) services among MSM and transgender women was very low, only 13% of MSM and 22% of transgender women had visited an HTC centre in the last 12 months.<br><br>Among MSM, the multiple logistic regression model revealed a negative association between having an income above 10,000 Nepalese rupees and condom use in the most recent sexual encounter.<br><br>Among transgender women, being a resident of the western region was positively associated with condom use in the last sexual intercourse.<br><br>Respondents that identified as transgender women and reported being away from home the past month were 2.4 more likely to have used condoms in their last sexual intercourse. | | | | | | | | | | | |
| 54 | Tuo et al. (2020) | 2020 | | Age, marital status, educational level, previous vaginal infection, drug use | L | < 30 | | U | | | Sex workers | | | |
| 55 | Upadhyay et al | 2020 | Socioeconomic status | | L | < 30 | R | | L | | U | 74% | | |
| 56 | Virdausi et al | 2022 | | | L | < 30 | | U | L | | Emp | 88.74 % | | 39.72% |
| 57 | Wangetal2021 | 2021 | - | | H | > 30 | | U | H | Y | Emp | | | |
| 58 | Wansometal2021 | 2021 | sexual attraction to mostly or only men, younger age, five or greater lifetime sexual partners, inconsistent condom use with | | H | < 30 | | | H | | Emp | | | |

| | | | | | | | | | | | | | | |
|---|---|---|---|---|---|---|---|---|---|---|---|---|---|---|
| | | | casual partners, and prior HIV testing | | | | | | | | | | | |
| 59 | Wilsonetal2021 | 2021 | - | | | | | | | | Sex workers | | | |
| 60 | Wuetal2019 | 2019 | - | | L | > 30 | | | | | Emp | H | | |
| 61 | Yan Et Al | 2021 | Educational level , Sexual activity | NA | | | | | | | | | 33.30% | 49.10% |
| 62 | Yi Et Al | 2018 | Knowledges , Attitudes | Age , Marital status , Educational status , Entertainment establishment , Monthly Income , Duration as a FEW in their respective workplace | H | > 30 | | U | | | sex workers | | | |
| 63 | Yu Et Al | 2020 | Educational level ( HIV knowledge ) , age , mobility , drug usage | Age group , sexual habit , educational level , length of work in current location | L | < 30 | | | | | sex workers | H | | |
| 64 | Zakaria et al | 2020 | | | | < 30 | | | H | Y | | H | | |
| 65 | Zarei et al | 2018 | Age, marital status, education, wealth, urban/rural areas | | H | < 30 | U | | H | | | L | | 15.40% |
| 66 | Zhou et al | 2020 | martial status, income, use of condoms/contraceptives in commercial sex, having STI symptoms | | L | < 30 | | M | L | | Sex workers | | | |
| 67 | Zin et al | 2019 | age | | L | < 30 | | | L | | | L | | 23.1/25 |
